# The Multiple Localized Glyceraldehyde-3-Phosphate Dehydrogenase Contributes to the Attenuation of the *Francisella tularensis dsbA* Deletion Mutant

**DOI:** 10.3389/fcimb.2017.00503

**Published:** 2017-12-11

**Authors:** Ivona Pavkova, Monika Kopeckova, Jana Klimentova, Monika Schmidt, Valeria Sheshko, Margarita Sobol, Jitka Zakova, Pavel Hozak, Jiri Stulik

**Affiliations:** ^1^Department of Molecular Pathology, Faculty of Military Health Science, University of Defence, Hradec Kralove, Czechia; ^2^Department of Biology of the Cell Nucleus, Institute of Molecular Genetics ASCR v.v.i., Prague, Czechia; ^3^Microscopy Centre–LM & EM, Institute of Molecular Genetics ASCR v.v.i., Prague, Czechia; ^4^Division BIOCEV, Laboratory of Epigenetics of the Cell Nucleus, Institute of Molecular Genetics ASCR v.v.i., Vestec, Czechia

**Keywords:** DsbA, SILAC, glyceraldehyde-3-phosphate dehydrogenase, *Francisella tularensis*, moonlighting

## Abstract

The DsbA homolog of *Francisella tularensis* was previously demonstrated to be required for intracellular replication and animal death. Disruption of the *dsbA* gene leads to a pleiotropic phenotype that could indirectly affect a number of different cellular pathways. To reveal the broad effects of DsbA, we compared fractions enriched in membrane proteins of the wild-type FSC200 strain with the *dsbA* deletion strain using a SILAC-based quantitative proteomic analysis. This analysis enabled identification of 63 proteins with significantly altered amounts in the *dsbA* mutant strain compared to the wild-type strain. These proteins comprise a quite heterogeneous group including hypothetical proteins, proteins associated with membrane structures, and potential secreted proteins. Many of them are known to be associated with *F. tularensis* virulence. Several proteins were selected for further studies focused on their potential role in tularemia's pathogenesis. Of them, only the gene encoding glyceraldehyde-3-phosphate dehydrogenase, an enzyme of glycolytic pathway, was found to be important for full virulence manifestations both *in vivo* and *in vitro*. We next created a viable mutant strain with deleted *gapA* gene and analyzed its phenotype. The *gapA* mutant is characterized by reduced virulence in mice, defective replication inside macrophages, and its ability to induce a protective immune response against systemic challenge with parental wild-type strain. We also demonstrate the multiple localization sites of this protein: In addition to within the cytosol, it was found on the cell surface, outside the cells, and in the culture medium. Recombinant GapA was successfully obtained, and it was shown that it binds host extracellular serum proteins like plasminogen, fibrinogen, and fibronectin.

## Introduction

Pathogenic bacteria produce a wide range of virulence factors whose activity, stability, and protease resistance depend on their correct folding through the formation of disulfide bonds between two cysteine thiol groups. The process of protein oxidative folding is achieved via the disulfide bond formation (Dsb) system and occurs within the periplasm in Gram-negative bacteria. The best characterized Dsb system is that of *Escherichia coli*. It is composed of two pathways: the oxidation pathway, with DsbA and DsbB proteins, and the isomerization pathway, with DsbC, DsbD, and DsbG proteins (Inaba, 2009). Inasmuch as there is growing evidence of extreme diversity in Dsb systems among bacteria (Bocian-Ostrzycka et al., 2015; Smith et al., 2016); however, the *E. coli* system should no longer be regarded as a universal model for bacteria. The key protein of this system—the non-specific disulfide oxidoreductase DsbA—introduces the disulfide bonds directly into extracytoplasmic proteins, including toxins, secretion systems, adhesins, or motility machines. Mutants with inactivated *dsbA* gene thus display attenuated virulence (Heras et al., 2009; Shouldice et al., 2011). Furthermore, a number of periplasmic proteins are also affected by the *dsbA* deletion, thereby reflecting the pleiotropic phenotype of relevant mutants.

*Francisella tularensis* is an intracellular, Gram-negative bacterium causing the zoonotic disease tularemia. Two *F. tularensis* subspecies are most associated with human disease: subsp. *tularensis* (type A) and subsp. *holarctica* (type B). Its low infectious dose, easy transfer, and extreme virulence cause *F. tularensis* to be a severe threat to human health, particularly because it can be misused as a bioterrorism agent. One gene of the *Francisella* genome encodes a protein with homology to DsbA: *Ft*DsbA. This also is referred to as *Francisella* infectivity potentiator protein B (FipB) by Qin et al. (2011). Its significant role in virulence has been demonstrated in numerous previously published studies. In several respects, *Ft*DsbA is unique from other known bacterial homologs. It is a glycosylated lipoprotein (Straskova et al., 2009; Thomas et al., 2011) with multiple functions, including not only oxidoreductase but also isomerase and chaperone activities (Straskova et al., 2009; Schmidt et al., 2013; Qin et al., 2014). It seems to be responsible for the introduction of disulfide bonds into various proteins, as well as for the repair of incorrectly formed disulfide bonds and protection of proteins from aggregation and misfolding. It is therefore assumed to influence the stability and function of diverse, predominantly extracytosolic proteins, many of which might directly mediate the *F. tularensis* virulence. Identification of proteins whose activity depends on DsbA can consequently reveal heretofore undetected virulence factors involved in molecular mechanisms of tularemia's pathogenesis. Accordingly, many of *Ft*DsbA's substrates identified in previously published analyses are known virulence factors. In our earlier study using two distinct comparative proteomic approaches (two-dimensional gel electrophoresis and shotgun iTRAQ analysis), we were able to identify only nine proteins with abundances different in a *dsbA* mutant strain compared to the wild-type strain of *F. tularensis* LVS (Straskova et al., 2009). Several of these were known virulence factors, such as DipA (Chong et al., 2013) or PdpE from the *Francisella* pathogenicity island (FPI) (Bröms et al., 2010). Two other studies applied more stringent trapping assays to look for the *Ft*DsbA substrates in *F. tularensis* strains with *dsbA* gene mutations in regions responsible for the substrate binding (Ren et al., 2014; Qin et al., 2016). These approaches enabled the identification of far more proteins requiring *Ft*DsbA for correct disulfide bond formation. Ren et al. (2014) found more than 50 *Ft*DsbA substrates, including a number of known virulence factors (DipA, FopA, MipA, type IV pili component FTL_0359, and two FPI proteins: PdpE and PdpB). In addition, two novel hypothetical proteins, FTL_1548 and FTL_1709, were shown to be required for *F. tularensis* virulence. Even more potential *Ft*DsbA substrates were identified by Qin et al. (2016). Again, several of them were known virulence factors. These include three FPI proteins (IglC, IglB, and PdpB). The marked discrepancy in number of proteins detected by our proteomic study compared to the trapping assays together with the availability of more advanced quantitative shotgun approaches led us to a decision to reanalyze the changes in membrane proteome induced by a lack of DsbA.

Here, we examined changes in membrane proteome caused by in-frame deletion of the *dsbA* gene in virulent type B strain of *F. tularensis* subsp. *holarctica* FSC200 (locus_tag FTS_1096). Using the highly sensitive stable isotope labeling with amino acids in cell culture (SILAC) technique, we succeeded in identifying 63 proteins with significantly altered abundance in the *dsbA* mutant strain. Fifteen proteins were further selected for elucidating their potential role in virulence, but only disruption of the gene encoding glyceraldehyde-3-phosphate dehydrogenase (GapA) resulted in attenuation of infection in the mouse model. Next, we provide a fundamental phenotypic characterization of the *gapA* deletion mutant strain, including experimental evidence of GapA's extracytosolic localization which provides convincing evidence of additional non-enzymatic functions of GapA in *F. tularensis*.

## Materials and methods

### Bacterial strains and culture

The *F. tularensis* and *E. coli* strains used in this study are listed and described in Table 1. All *F. tularensis* strains were cultured on McLeod agar enriched with bovine hemoglobin (Becton Dickinson, Cockeysville, MD, USA) and IsoVitaleX (Becton Dickinson) and in liquid Chamberlain's medium at 37°C (Chamberlain, 1965), supplemented with tryptone (10 mg/mL) when indicated. *E. coli* strains were grown on Luria–Bertani (LB) agar and in LB broth at 37°C. Where appropriate, antibiotics were used at the following concentrations: chloramphenicol 2.5 μg/mL (*F. tularensis*) or 25 μg/mL (*E. coli*), polymyxin B 75 μg/mL, ampicillin 100 μg/mL, and penicillin 100 U/mL.

**Table 1:** Bacterial strains and plasmids used in this study.

**Strain/Plasmid**	**Genotype/Phenotype**	**Source/References**
***F. tularensis***		
FSC200	*F. tularensis* subsp. *holarctica*; clinical isolate	*Francisella* strain collection Johansson et al., 2000
dsbA	ΔFTS_1067/FSC200	Straskova et al., 2009
gapA	ΔFTS_1117/FSC200	This study
gapA-complemented	ΔFTS_1117/FSC200::FTS_1117	This study
***E. coli***		
Top10	F^−^*mcrA* Δ(*mrr-hsdRMS-mcrBC*), ϕ80*lacZ*ΔM15 Δ*lacX74 recA1 deoR araD139* (Δ*ara-leu*)*7697 galU galK rpsL* (Sm^r^) *endA1 nupG*	Invitrogen
S17-1λpir	*recA, thi, pro, hsdR*^−^*M*+, <RP4:2-Tc:Mu:Km:Tn*7*>Tp^R^, Sm^R^	Simon et al., 1983
**Plasmids**		
pCR®4.0-TOPO	TOPO-cloning vector. Amp^R^, Km^R^	Invitrogen
pDM4	Suicide plasmid. sacB; mobRP4; oriR6K; Cm^R^	Milton et al., 1996

### Construction of in-frame deletion mutant *gapA* and complementation

The DNA construct encoding in-frame deletion for the *gapA* gene with introduced restriction sites XhoI and SpeI (locus tag FTS_1117) was generated by overlapping PCR amplification using the primers A–D shown in Table 2. The resulting DNA fragment was cloned into pCR4-TOPO vector (Invitrogen, Carlsbad, CA, USA) and verified by sequence analysis (ABI PRISM 3130xl, Applied Biosystems). Fragments from plasmids with verified inserts were cloned into pDM4 (Table 1), then introduced into *E. coli* S17-1γpir. The in-frame deletion mutant *gapA* was complemented *in cis* using a strategy similar to the in-frame deletion mutagenesis described above. Preparation of plasmid DNA, restriction enzyme digests, ligations, and transformations into *E. coli* all were performed essentially as described (Sambrook and Russel, 2001).

**Table 2:** Sequences of primers used for creation of *F. tularensis* FSC200 *gapA* deletion mutant.

**Gene**	**Primer designation**	**5′–3′ Sequence**
gapA (FTS_1117)	A	*CTCGAG*TATATAGCTTCGCAATTGAGTAA
	B	GCTCCGAAGTAACCATTAATTGCAACTCTCATTTT
	C	GCAATTAATGGTTACTTCGGAGCTCTATAAACA
	D	*ACTAGT*ATCTCATCCGCAACAACATAG

*Restriction sites for selected endonucleases on primers A and D are in italic; complementary parts of primer B and C are underlined*.

### Proteomics

#### Metabolic labeling, membrane fraction preparation

*F. tularensis* was cultivated in Chamberlain's chemically defined medium (Chamberlain, 1965). The wild-type strain FSC200 was cultivated in the heavy variant of the medium containing isotopically labeled L-arginine hydrochloride [^13^C_6_^15^N_4_] and L-lysine hydrochloride [^13^C_6_^15^N_2_] (Sigma-Aldrich, Schnelldorf, Germany) in the same concentrations as in the light medium. The *dsbA* deletion mutant strain was grown in the light medium. Bacteria were cultivated in the corresponding media overnight at 36.8°C, 200 rpm. Overnight cultures were diluted 20 times with fresh media and again grown overnight. Bacteria were then pelleted, diluted with fresh media to OD_600 nm_ 0.1 and cultivated to OD_600 nm_ 0.8 (late exponential phase). Three biological replicates were prepared. Harvested and washed bacteria were suspended in phosphate-buffered saline (PBS, pH 7.4) supplemented with protease inhibitor cocktail Complete EDTA-free (Roche Diagnostics, Germany) and benzonase (150 U/mL, Sigma-Aldrich), then disrupted in a French press (Thermo IEC, Needham Heights, MA, USA) by three passages at 16,000 PSI. Unbroken cells were removed by centrifugation at 6,000 × g for 30 min at 4°C. Protein concentration was determined using a bicinchoninic acid protein assay kit (Sigma-Aldrich). Corresponding light and heavy cell lysates were mixed in a 1:1 protein ratio and centrifuged at 120,000 × g for 30 min at 4°C in a Beckman TLA-100.3 fixed angle rotor (Beckman Instruments, Palo Alto, CA, USA). Supernatants were discarded and pellets (membrane-enriched fractions) were suspended in 50 mM Tris/HCl, pH 8.0, and pelleted again. Pellets were stored at −80°C until the mass spectrometry (MS) analysis.

#### Protein digestion for mass spectrometry analysis

The membrane pellets were dissolved in 50 mM ammonium bicarbonate with 5% (w/v) sodium deoxycholate (SDC), then incubated for 20 min on ice with intermittent vortexing. Protein concentration was then determined. Samples were then reduced with 10 mM dithiothreitol at 37°C for 60 min, alkylated with 20 mM iodoacetamide at room temperature for 30 min in darkness, and the unreacted iodoacetamide was quenched with additional 10 mM dithiothreitol at room temperature for 15 min. The samples were diluted with 50 mM ammonium bicarbonate to reduce the concentration of SDC to 0.5% and digested with sequencing grade trypsin (Promega, Madison, WI, USA) overnight at 37°C. SDC was removed using the modified phase transfer protocol (Masuda et al., 2008). Briefly, ethyl acetate was added and the digested product was acidified by trifluoroacetic acid (TFA) to a final concentration of ca 2% (v/v). The mixtures were vortexed vigorously for 1 min, centrifuged at 14,000 × g for 5 min, and the upper organic layer was removed. The extraction was repeated with a fresh portion of ethyl acetate. The aqueous phases were desalted on Empore™ C18-SD (4 mm/1 mL) extraction cartridges (Sigma-Aldrich) and dried in a vacuum.

#### Peptide separation and mass spectrometry analysis

Liquid chromatography–mass spectrometry analysis was performed using the Ultimate 3000 RSLCnano System (Dionex, Sunnyvale, CA, USA) coupled online via a Nanospray Flex ion source with a Q-Exactive mass spectrometer (Thermo Scientific, Bremen, Germany). Peptide mixtures were dissolved in 2% acetonitrile/0.05% TFA and 500 ng of each was loaded onto a capillary trap column (C18 PepMap100, 3 μm, 100Å, 0.075 × 20 mm; Dionex) using 5 μL/min of 2% acetonitrile/0.05% TFA for 5 min. They were then separated on a capillary column (C18 PepMap RSLC, 2 μm, 100Å, 0.075 × 150 mm; Dionex) using a step linear gradient of mobile phase B (80% acetonitrile/0.1% formic acid) over mobile phase A (0.1% formic acid) from 4 to 34% B in 68 min and from 34 to 55% B in 21 min at flow rate of 300 nL/min. The column was kept at 40°C and the eluent was monitored at 215 nm. Spraying voltage was 1.75 kV and heated capillary temperature was 200°C. The mass spectrometer operated in the positive ion mode performing survey MS (at 350–1,650 m/z) and data-dependent MS/MS scans of the 10 most intense precursors with a dynamic exclusion window of 60 s and isolation window of 2.0 Da. MS scans were acquired with resolution of 70,000 from 3 × 10^6^ accumulated charges; maximum fill time was 100 ms. Normalized collision energy for higher-energy collisional dissociation (HCD) fragmentation was 27 units. MS/MS spectra were acquired with resolution of 17,500 from 10^5^ accumulated charges; maximum fill time was 100 ms. Each biological replicate was analyzed three times (total of nine measurements).

#### Protein identification and quantification

Database search and relative protein quantification were performed using Proteome Discoverer 1.3 (v. 1.3.0.339, Thermo Scientific) and the Mascot search algorithm. The reference proteome set of *F. tularensis* FSC200 was downloaded from UniProt/KB in March 2013 and merged with a common contaminants file downloaded from the MaxQuant web page (http://www.maxquant.org/downloads.htm). The merged database contained 1,671 sequences. The search parameters were as follows: digestion with trypsin, maximum two missed cleavages allowed, peptide mass tolerance of 10 ppm, fragment mass tolerance of 0.02 Da, fixed carbamidomethylation of cysteine, variable oxidation of methionine, and SILAC labels Arg10 and Lys8. The strict target value of false discovery rate (FDR) for a decoy database search of 0.01 was applied (high confidence). Only unique peptides were considered for quantification and the light–to–heavy ratios were normalized on protein median for each replicate. The quantification workflow was performed on two levels. On the first level, missing quantitative values were not considered and the maximum allowed fold change was set to 100. In the second level, extreme ratios were considered, the missing quantitative values were replaced by the minimum value detected (the detection threshold) and ratios above the maximum allowed fold change were used for quantification. The second-level quantification allowed for quantification of additional proteins (highlighted by asterisk in Table 3).

**Table 3:** Proteins with significantly altered expression in *dsbA* mutant compared to the FSC200 wild-type strain detected by SILAC quantitative shotgun approach.

**Accession^a^**	**FTS locus tag**	**Protein name^b^**	**up-/down-^b^**	**COG^c^**	**References**
AFT93177	1495	Hypothetical protein, FTS_1495	up-	S	Straskova et al., 2009; Konecna et al., 2010; Ren et al., 2014; Qin et al., 2016
AFT93208	1538	Hypothetical protein FTS_1538	up-	S	Straskova et al., 2009; Konecna et al., 2010; Pávková et al., 2013; Ren et al., 2014
AFT93168	1485	Chitinase family 18 protein	up-	G	Kadzhaev et al., 2009; Straskova et al., 2009; Chung et al., 2014; Ren et al., 2014
AFT93368	**1749**	**Hypothetical protein FTS_1749**	up-	S	Ren et al., 2014; Qin et al., 2016
AFT92137	0123	Pyruvate phosphate dikinase	up-	G	
AFT92841	1034	D-alanyl-D-alanine carboxypeptidase	up-	M	Straskova et al., 2009; Ren et al., 2014; Qin et al., 2016
AFT92421	**0495**	**Hypothetical protein FTS_0495**	up-	S	Ren et al., 2014
AFT93021	1279	Hypothetical protein FTS_1279	up-	S	Straskova et al., 2009; Chong et al., 2013; Ren et al., 2014; Qin et al., 2016
AFT93404	1789	Siderophore biosynthesis protein	up-	P	Ramakrishnan et al., 2008; Pérez et al., 2016
AFT93162	**1476**	**Glycerophosphoryl diester phosphodiesterase**	up-	I	Konecna et al., 2010
AFT92351	**0402**	**Hypothetical protein FTS_0402**	up-	S	Ren et al., 2014
AFT92903	**1117**	**Glyceraldehyde-3-phosphate dehydrogenase/erythrose-4-phosphate dehydrogenase**	up-	G	Konecna et al., 2010; Qin et al., 2016; this study
AFT93160	1471	Catalase/peroxidase	up-	P	Lindgren et al., 2007; Qin et al., 2016
AFT93076	1361	Parvulin-like peptidyl-prolyl isomerase domain-containing protein	up-	O	Su et al., 2007
AFT92667	0815	Hypothetical protein FTS_0815	up-	S	Ren et al., 2014
AFT92381	0450	Hypothetical protein FTS_0450	up-	S	Brotcke et al., 2006; Brotcke and Monack, 2008; Wehrly et al., 2009
AFT92582	0702	FAD binding family protein	up-	C	Ren et al., 2014
AFT92713	0868	X-prolyl aminopeptidase 2	up-	E	Guina et al., 2007
AFT93339	1709	Elongation factor Tu	up-	J	Barel et al., 2008; Qin et al., 2016
AFT92546	**0659**	**Hypothetical protein FTS_0659**	up-	S	Straskova et al., 2009
AFT92986	**1229**	**Hypothetical protein FTS_1229**	up-	S	
AFT92133	0111/1139	Hypothetical FTS_0111	up-	S	Robertson et al., 2013; Bröms et al., 2016
AFT92684	0836	Isochorismatase hydrolase family protein	up-	Q	Pavkova et al., 2006; Su et al., 2007
AFT92956	1187	Hypothetical protein FTS_1187	up-	S	Wehrly et al., 2009; Qin et al., 2016
AFT92755	**0920**	**Hypothetical protein FTS_0920**	up-	S	Brotcke et al., 2006
AFT92560	**0676**	**Hypothetical protein FTS_0676**	up-	S	
AFT92796	**0974**	**Hypothetical protein FTS_0974**	up-^*^	S	Ren et al., 2014
AFT92765	**0935**	**GTP-dependent nucleic acid-binding protein EngD**	up-^*^	J	
					
AFT92195	0200	Hypothetical protein FTS_0200	down-	S	Dieppedale et al., 2011, 2013
AFT92865	1068	Hypothetical protein FTS_1068	down-	S	Straskova et al., 2009; Qin et al., 2011
AFT92060	0012	Inhibitor of RecA	down-	L	
AFT92481	**0580**	**Sugar porter (SP) family protein**	down-	G	
AFT93103	1397	Glycosyltransferase family protein	down-	M	Bandara et al., 2011
AFT92194	0199	von Willebrand factor type A domain-containing protein	down-	R,O	Dieppedale et al., 2011, 2013
AFT92732	0890	Protease, GTP-binding subunit	down-	J	Su et al., 2007
AFT92334	0381	Type IV pili, pilus assembly protein	down-	N,W	Salomonsson et al., 2011
AFT93371	1752	FOF1 ATP synthase subunit gamma	down-	C	
AFT92172	0175	LPS fatty acid acyltransferase	down-	M	McLendon et al., 2007
AFT92106	0079	Acyltransferase	down-	I	
AFT92933	1158	Ribonuclease HII	down-	L	Kadzhaev et al., 2009
AFT93108	1402	ABC transporter ATP-binding protein	down-	V	Dankova et al., 2016
AFT92145	0137	Hypothetical protein FTS_0137	down-	S	
AFT93370	1751	FOF1 ATP synthase subunit beta	down-	C	Qin et al., 2016
AFT93375	1756	F0F1 ATP synthase subunit C	down-	C	
AFT93243	1582	Drug: H+ antiporter-1 (DHA1) family protein	down-	V	Su et al., 2007
AFT93130	**1439**	**Excinuclease ABC, subunit A**	down-	L	Su et al., 2007; Kadzhaev et al., 2009
AFT92197	0202	Hypothetical protein FTS_0202	down-	S	Dieppedale et al., 2011, 2013
AFT93269	1620	Nucleoside permease NUP family protein	down-	F	
AFT92502	0602	O-antigen flippase	down-	C	Dankova et al., 2016
AFT92502	1754	FOF1 ATP synthase subunit delta	down-	C	
AFT93369	1750	FOF1 ATP synthase subunit epsilon	down-	C	
AFT93467	1882	ATP-binding cassette (ABC) superfamily protein	down-	P,R	Asare and Abu Kwaik, 2010
AFT92984	1226	ATP-dependent RNA helicase	down-	L	
AFT93372	1753	FOF1 ATP synthase subunit alpha	down-	C	Qin et al., 2016
AFT92658	0799	Amino acid transporter	down-	E	Kadzhaev et al., 2009
AFT92817	1004	Radical SAM superfamily protein	down-	J	
AFT92323	**0367**	**Hypothetical protein FTS_0367**	down-	S	
AFT92497	0597	Membrane protein/O-antigen protein	down-	M	Kim et al., 2010
AFT93227	1562	Delta-aminolevulinic acid dehydratase	down-	H	
AFT92449	0533	Uracil-DNA glycosylase	down-	L	
AFT93102	1396	Glycosyltransferases group 1 family protein	down-	M	Weiss et al., 2007; Bandara et al., 2011; Thomas et al., 2011
AFT92105	**0078**	**Acyltransferase**	down-^*^	I	
AFT92192	0197	Uncharacterized protein FTS_0197	down-^*^	S	Dieppedale et al., 2011, 2013

a *Accession numbers and protein names according to NCBI (https://www.ncbi.nlm.nih.gov)*.

b Up- or down-regulated proteins according to the following criteria: statistical significance p < 0.05; relative changes ≥ 1.5 for up-regulated or ≤ for down regulated proteins.

* *Quantified by second-level quantification workflow (extreme ratios considered)*.

c *Predicted function of proteins by COG using the COGnitor program (http://www.ncbi.nlm.nih.gov/COG/). S, no functional prediction; G, carbohydrate metabolism and transport; M, cell wall structure and biogenesis and outer membrane; P, inorganic ion transport and metabolism; I, lipid metabolism; O, molecular chaperons and related functions; C, energy production and conversion; E, amino acid metabolism and transport; J, translation, ribosome structure, and biogenesis; N, secretion, motility, and chemotaxis; W, extracellular structure; L, replication, recombination, and repair; V, defense mechanisms; F, nucleotide transport and metabolism; Q, secondary metabolites biosynthesis, transport, and catabolism; H, coenzyme transport and metabolism; R, general function prediction only*.

*Genes encoding proteins highlighted in bold were inactivated using retargeted mobile group II introns and tested for potential attenuation in vivo*.

For relative protein quantification, only proteins with minimum of 1 quantified peptide per at least 6 measurements out of 9 were considered. Light–to–heavy ratios were converted to log_2_ and median was determined from the three measurements for each biological replicate. Mean, standard deviation, and the *t*-statistic *p*-value were then evaluated from the three biological replicates. For further study, proteins showing absolute change larger than 1.5 and *p* ≤ 0.05 were considered (see Table 3 and Supplementary Table 2). The mass spectrometry proteomics data have been deposited to the ProteomeXchange Consortium via the PRIDE (Vizcaíno et al., 2016) partner repository with the dataset identifier PXD007682.

### Intracellular replication and invasion assays

To generate bone marrow macrophages (BMMs), bone marrow cells were collected from dissected femurs of female BALB/c mice 6–10 weeks old and differentiated into macrophages in Dulbecco's Modified Eagle Medium (DMEM, Invitrogen) supplemented with 10% fetal bovine serum and 20% L929-conditioned medium for 6–7 days (Celli, 2008). The differentiated BMMs were seeded at concentration 5 × 10^5^ cells/well in 24-well plates and infected the next day with *F. tularensis* strains at multiplicity of infection (MOI) 50:1 (bacteria/cell). To synchronize the infection, the infected cells were centrifuged at 400 × g for 5 min and incubated at 37°C for 30 min. The extracellular bacteria were then removed by gentamicin treatment (5 μg/mL) for 30 min. For the proliferation assay, the infected BMMs were lysed at selected time points with 0.1% SDC. To determine the number of intracellular bacteria, the lysates were serially diluted and plated on McLeod agar. The human alveolar type II epithelial cell line A549 (ATCC® CCL-185™) was cultured in DMEM supplemented with 10% fetal bovine serum (Invitrogen). Infections were carried out as described by Lo et al. (2013). Briefly, the cells were infected with *F. tularensis* strains at MOI of 200 and incubated for 3 h. The extracellular bacteria were removed by gentamicin treatment, the cells were lysed at selected time points, then the cells were plated in serial dilution on McLeod agar.

### Cell viability and cytotoxicity assay

To follow the viability and cytotoxic effect of *F. tularensis* strains on BMMs, the cells were seeded in 96-well tissue culture plates at concentration 3 × 10^4^ cells/well and allowed to adhere overnight. The next day, the BMMs were infected with bacterial cell suspensions at a MOI of 100:1 for 1 h. The extracellular bacteria were washed thoroughly away and the cells were incubated in DMEM medium at 37°C with 5% CO_2_. The cell viability was observed in real time using a non-lytic bioluminescence RealTime-Glo™ MT Cell Viability Assay (Promega, Madison, WI, USA). The cytotoxicity was assayed in parallel using fluorescence CellTox™ Green Cytotoxicity Assay (Promega). The samples were processed and the luminescence and fluorescence were measured at indicated time points on a FLUOStar OPTIMA plate reader (BMG Labtech, Germany) according to the manufacturer's instructions.

### Animal studies

For survival studies, groups of at least five female BALB/c mice 6–8 weeks old were infected subcutaneously (s.c.) with the *gapA* mutant strain (using doses of 1.5 × 10^2^, 3 × 10^2^, 3 × 10^5^, and 3 × 10^7^ cfu/mouse), the wild-type FSC200 strain (wt strain), and *gapA*-complemented strain (both at 10^2^ cfu/mouse). Control groups of mice were inoculated with sterile saline only. Mice were housed in micro-isolator cages and fed sterilized water and food *ad libitum*. Infected mice were examined every day for signs of illness through 42 days. For protection studies, the mice were challenged s.c. 42 days post-infection with 3 × 10^2^ cfu of the wt strain and monitored for survival for an additional 21 days. Three independent experiments were performed. For growth kinetics studies, groups of three BALB/c mice were infected with 3 × 10^2^ cfu/mouse of wt strain, *gapA* mutant strain, or *gapA*-complemented strain. At 1, 3, 5, 7, 14, 21, 28, 35, and 42 days after infection, spleens and livers were aseptically removed, homogenized in 2 mL of PBS, and serial dilutions plated onto McLeod agar.

### Preparation of the whole-cell lysate, crude membrane fraction, and culture filtrate proteins

Bacteria were grown in Chamberlain's medium at 37°C until the late logarithmic phase (0.7–0.8 O.D.). To obtain whole-cell lysate, the bacteria were lysed in a French pressure cell and unbroken cells and cell debris were removed by centrifugation. The pellet with membrane proteins was obtained by ultracentrifugation of the whole-cell lysate, then suspended in PBS as described previously. For the preparation of culture filtrate proteins (CFP), the bacteria were removed by centrifugation and the culture medium was vacuum-filtered through membranes (0.2 μm pore; Merck Millipore, Billerica, MA, USA). The filtrate was then concentrated using Stirred Ultrafiltration Cell (8200, Millipore) with 5 kDa cut-off membrane from regenerated cellulose (Millipore) followed by diafiltration using Amicon Ultra 3K devices (Millipore) to further concentrate the protein sample and exchange the medium for 40 mM Tris/HCl (pH 7.3). The protein content was determined using a bicinchoninic acid assay.

### 2D gel electrophoresis and immunodetection

Protein samples (150 μg) were dissolved in rehydration buffer containing 1% (w/v) ASB-14 surfactant. Isoelectric focusing in the non-linear pH range of 3–10 and gradient 9–16% SDS-PAGE in the second dimension were performed as described previously (Straskova et al., 2009). Separated proteins were electroblotted onto polyvinylidene difluoride membranes and the GapA protein was detected using a polyclonal rabbit anti-GapA antibody (Apronex, Vestec, Czech Republic). Swine anti-rabbit IgG/HRP (Dako, Glostrup, Denmark) was applied as secondary antibody. Chemiluminescence was detected using a BM Chemiluminescence Blotting Substrate (POD) according to the manufacturer's instructions (Roche Diagnostics, Mannheim, Germany).

### Transmission electron microscopy

The overnight culture of wild-type strain bacteria (FSC200) grown in Chamberlain's medium was pelleted, washed in PBS, then fixed in a mixture of 3% formaldehyde plus 0.1% glutaraldehyde in 0.1 M Na/K phosphate buffer, pH 7.2–7.4 (Sörensen's buffer) in the ratio of 10:1 (v:v) for 30 min at room temperature. The fixed bacteria were centrifuged for 15 min at 7,300 rpm and the pellet was twice washed in ice-cold Sörensen's buffer. The pellet was then mixed with 2.5% agarose and centrifuged (25 min, 8,500 rpm, +37°C). The free aldehyde groups were quenched in 0.02 M glycine in Sörensen's buffer for 10 min. The sample was washed in Sörensen's buffer two times for 7 min each time. The samples were separated into two groups and processed for either LR White or Lowicryl HM20 resin embedding. “LR White” samples were dehydrated in 30, 50, 70, 90, and 96% ethanol for 7 min each, infiltrated in the mixtures of LR White with 96% ethanol (1:2 and 2:1 for 30 min each), then placed into LR White for overnight infiltration. All steps were carried out at +4°C. The next day, the samples were infiltrated in fresh LR White for 3 h at +4°C and polymerized by UV for 48 h at +4°C. “Lowicryl HM20” samples were dehydrated in 30% (30 min, +4°C), 50% (1 h, −20°C), 70% (1 h, −35°C), and 100% ethanol (two times for 1 h each time, −50°C), infiltrated in the mixtures of Lowicryl HM20 with 100% ethanol (1:1 and 2:1 for 1 h each, −50°C), placed into Lowicryl HM20 for 1 h (−50°C), then into fresh resin for overnight infiltration at −50°C. The next day, the temperature was elevated to −35°C and the samples were UV-polymerized for 24 h at −35°C and 72 h at room temperature.

Ultrathin sections (70 nm) were immunolabeled following a conventional protocol (Strádalová et al., 2008) and examined using Morgagni 268 (at 80 kV) and Tecnai G2 20 LaB6 (at 200 kV) transmission electron microscopes (FEI, Eindhoven, The Netherlands). The images were captured with Mega View III CCD and Gatan Model 894 UltraScan 1000 cameras. Multiple sections of repetitive immunogold labeling experiments were analyzed. Antibodies used for immunolabeling were primary rabbit anti-FTT antibody (dilution 1:25 to 1:100) and secondary goat anti-rabbit IgG (H + L) antibody coupled with 12 nm colloidal gold particles (Jackson ImmunoResearch Laboratories Inc., 111-205-144; dilution 1:30).

### Expression and purification of recombinant gapA protein

PCR was used to amplify the *F. tularensis gapA* gene with specific primers containing an NcoI site in the forward primer (5′-CCCCATGGGTTTTAATAAACTTTCGCAAGATAA-3′) and an XhoI site in the reverse primer (5′-GGCTCGAGTAGAGCTCCGAAGTACTCT-3′). The gel-purified PCR products (Qiagen) were cloned into pET28b, resulting in the recombinant plasmid encoding GapA with a C-terminal histidine tag. The plasmid construct was verified by direct DNA sequencing. Expression vector pET-*gapA* was transformed into *E. coli* NiCo214(DE3)(NEB) cells for production and purification of recombinant protein. Cells were grown at 37°C to A600 of 0.6, and expression was subsequently induced using 1 mM IPTG for 4 h. Pelleted cells were lysed by sonication in lysis buffer (50 mM phosphate, 300 mM NaCl). The cell extract was clarified at 20,000 rpm for 30 min at 4°C, and supernatant was applied to a TALON column (Clontech) for purification of His-tagged GapA. Eluted fractions were verified by SDS-PAGE followed by Coomassie staining and immunoblotting for the presence of GapA.

### Glyceraldehyde-3-phosphate dehydrogenase (GAPDH) activity assay

GAPDH activity measurement was conducted according to Pancholi and Fischetti (1992). The purified recombinant protein, whole-cell lysate, or fraction enriched in CFPs were mixed with 7 μL glyceraldehyde-3-phosphate (50 mg/mL, substrate), 100 μL NAD^+^ (10 mM), and assay buffer (40 mM triethanolamine, 50 mM Na_2_HPO_4_, and 5 mM EDTA, pH 8.6) in a final volume of 1 mL. Negative control assays were performed without the addition of substrate. The conversion of NAD^+^ to NADH was monitored spectrophotometrically at 340 nm at regular time intervals for a given time.

### Binding assays of GapA to selected human proteins

#### Far-western assay

This method used for detection of GapA binding to human proteins fibrinogen, fibronectin, actin, and plasminogen was performed according to Egea et al. (2007). The proteins (5 μg each, Sigma-Aldrich) were separated on a 12% SDS-PAGE and transferred to a PVDF membrane. The membrane was blocked in a solution of PBST [4 mM KH_2_PO_4_, 16 mM Na_2_HPO_4_, 115 mM NaCl (pH 7.4), 0.05% (v/v) Tween-20] and 5% (w/v) powdered milk for 1 h at room temperature. The membrane was incubated overnight with a purified recombinant GapA (0.5 μg/mL) diluted in 10 mL binding buffer [100 mM NaCl, 20 mM Tris (pH 7.6), 0.5 mM EDTA, 10% (v/v) glycerol, 0.1% (v/v) Tween-20, 2% (w/v) powdered milk, 1 mM dithiothreitol] at 4°C and washed three times in the PBST. To visualize the interaction between GapA and selected proteins, the membrane was incubated with anti-GapA antibody diluted 1:1,000 in PBST with 5% (w/v) powdered milk for 16 h at 4°C and further processed using the ECL Select Western Blotting Detection Reagent according to the manufacturer's instructions (GE Healthcare Life Sciences).

#### Solid-phase ligand-binding assay

The human proteins (plasminogen, fibrinogen, fibronectin, and actin) (0.5 μg/mL) diluted in 100 μL PBS [4 mM KH_2_PO_4_, 16 mM Na_2_HPO_4_, 115 mM NaCl (pH 7.4)] were coated on 96-well microtiter plate overnight at room temperature. PBS buffer was changed for TBS blocking buffer [20 mM Tris-HCl, 150 mM NaCl, (pH 7.6)] with 1% (w/v) powdered bovine serum albumin and incubated overnight at 4°C. The wells coated with proteins were incubated with purified recombinant GapA (0.125 μg/mL to 2 μg/mL) in TBS buffer with 1% bovine serum albumin for 3 h at room temperature. The plate was washed three times in TBS with 0.05% (v/v) Tween-20 and once in TBS buffer. The amount of GapA bound to these proteins was determined spectrophotometrically (450 nm) in an ELISA-based assay using anti-GapA antibody (1:10,000) followed by peroxidase-labeled donkey anti-rabbit anti-body (1:4000) and 3, 3′, 5, 5′-tetramethylbenzidine (Sigma-Aldrich) as chromogenic substrate.

### Ethics statement

This study was carried out in accordance with the recommendations of the guidelines of the Animal Care and Use Ethical Committee of the Faculty of Military Health Sciences, University of Defence, Czech Republic. The protocol was approved by the Ethical Committee of the Faculty of Military Health Sciences, University of Defence, Czech Republic.

### Statistical analysis

All the experiments were performed at least three times and each sample in each separate experiment was processed at least in triplicate. Values are expressed as mean ± standard deviation (*SD*) and analyzed for significance using Student's two-tailed *t*-test. Differences were considered statistically significant at *p* < 0.05.

## Results

### Semiquantitative analysis of membrane-enriched fractions of the *dsbA* mutant and the wild-type strain of subsp. *holarctica* FSC200. construction of selected TargeTron insertion mutants and their testing for *in vivo* attenuation

In our previously published study (Straskova et al., 2009), using the combination of classical gel-based and iTRAQ quantitative shotgun proteome analyses, we had been able to identify only nine proteins with significantly altered abundance in membrane-enriched fractions of the *dsbA* mutant strain compared to its wild counterpart. Inasmuch as the DsbA protein of *F. tularensis* demonstrably plays a role in proper folding of other proteins, this number was evidently underestimated. That is why we decided to analyze and compare the two membrane proteomes (*dsbA* mutants strain vs. wt strain) using SILAC metabolic labeling. This approach enabled us to find a total of 63 proteins that were either significantly more (30) or less (35) abundant in response to deletion of the *dsbA* gene (Table 3). Therefore, in contrast to our previous study, the number of known DsbA-regulated proteins was significantly enlarged. This confirms the high sensitivity of the applied proteomic approach.

According to the Cluster of Orthologous Genes (COG) categories classification, the *dsbA* gene deletion resulted in broad changes within the bacterial proteome that included proteins from a diverse set of functional categories (Supplementary Figure 3). The categories most represented involved poorly characterized proteins with unknown function (21) and proteins engaged in diverse metabolic processes (22). As seen in Table 3, most of the proteins affected by the *dsbA* gene deletion have previously been mentioned in the literature. Many of them are well-known virulence determinants; others have not been proven to play a direct role in the pathogenicity of tularemia infection. For the preliminary screening of new potential virulence factors dependent on DsbA oxidative folding, we successfully inactivated 15 genes encoding selected proteins (highlighted in bold in Table 3) using retargeted mobile group II introns as described previously (Rodriguez et al., 2009). The main criteria for the protein selection included lack of previously published data, known immunoreactive proteins (Kilmury and Twine, 2011; Chandler et al., 2015), and any evidence for association with virulence based on bioinformatics screening using NCBI or UniProt. All the data concerning the TargeTron insertion mutants, methodology, and results are summarized in the Supplementary Material. The prepared mutant strains were examined for their virulence potential in mice. Unfortunately, none of the tested mutant strains except for the strain with a targeted gene for glyceraldehyde-3-phosphate dehydrogenase (GapA, FTS_1117) revealed any signs of attenuation, as, similarly to the wt strain, all the challenged mice succumbed to infection around the 5th day. On the contrary, all the mice infected with the *gapA* insertion mutant (*gapA*in) in doses of 10^2^, 10^5^, and 10^7^ CFU/mouse survived the 21st day of infection. Furthermore, the proliferation of *gapAin* mutant was significantly reduced both within the murine bone-marrow derived macrophages and monocyte-macrophage cell line J774.2 (Supplementary Figures 1A,B). However, the mutant strain revealed a significant growth defect also in Chamberlain's chemically defined medium (Supplementary Figure 2) which can contribute to the diminished replication and *in vivo* attenuation.

### Preparation of in-frame deletion *gapA* mutant

The *gapA* gene seems to be a part of a five-gene operon encoding genes of the glycolytic/gluconeogenic pathways. Therefore, the observed attenuation in the *gapAin* mutant may be partly due to the polar effect on downstream genes as the transposons are in general known to cause polar mutations, thereby preventing transcription of downstream genes in an operon. That is why we decided to target the gene by in-frame deletion mutagenesis. GAPDH plays a key role in glucose metabolism, and its gene is thus essential in many bacteria. We nevertheless succeeded in constructing an *F. tularensis* viable *gapA* deletion mutant strain. Additionally, a complementation mutant strain was prepared and used in most of the experiments to ensure the observed phenotype is really due to elimination of the *gapA* gene.

#### The *gapA* deletion mutant is viable, retained the ability to enter the host cells but revealed a growth defect *in vitro*

**Figure 1:**
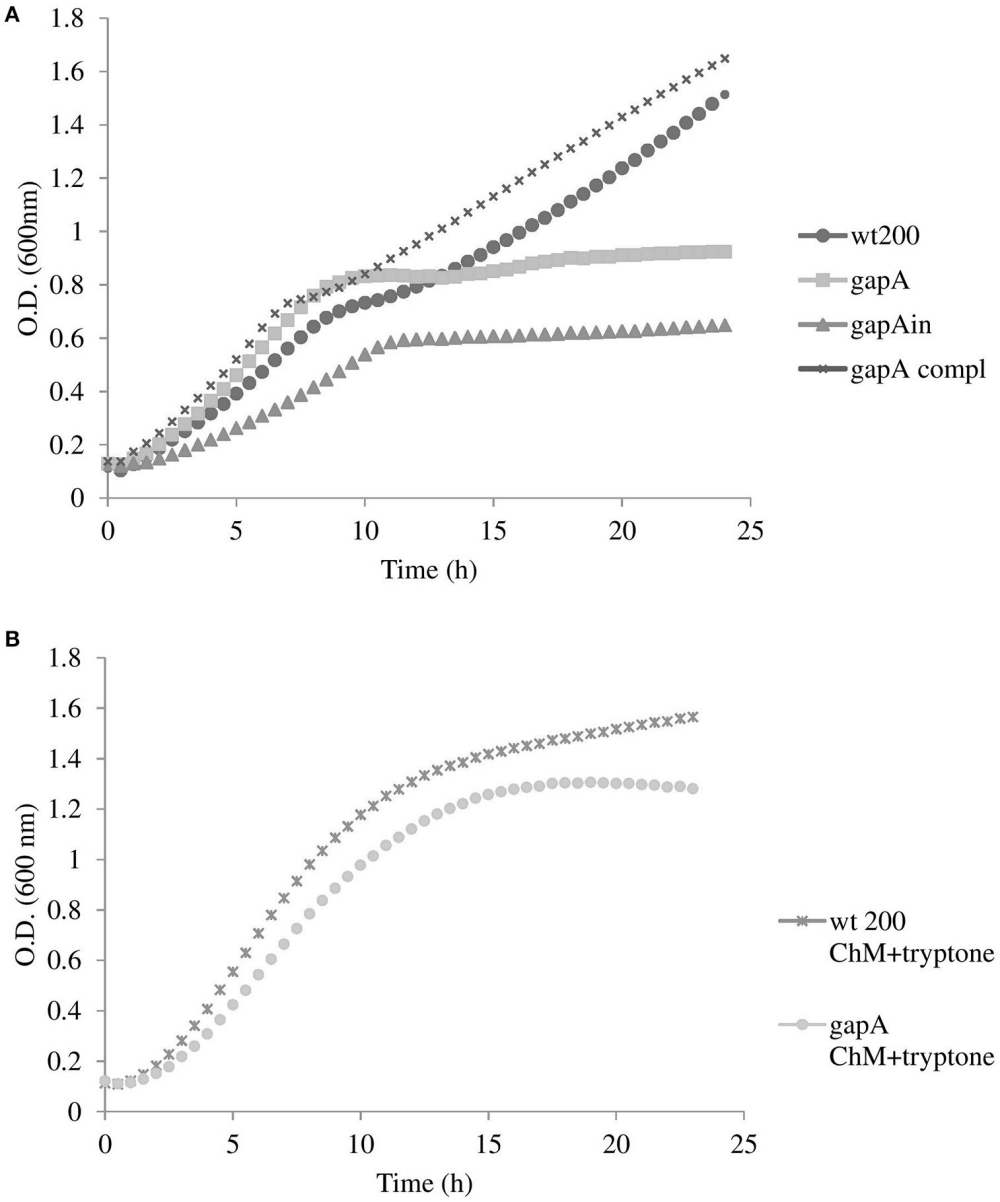
Growth curves for *F. tularensis* strains FSC200 wt, *gapA, gapA* complemented, and *gapA*in in Chamberlain's medium at 37°C **(A)**, and FSC200 wt and *gapA* strains in Chamberlain's medium supplemented with tryptone **(B)**. Bacterial growth was determined by measuring the OD_600 nm_ every 30 min in pentaplicate for 24 h. Three independent experiments were performed.

We first tested the effect of *gapA* deletion on the mutant's growth in a chemically defined Chamberlain's medium. As seen in Figure 1A, growth of the *gapA* mutant strain was comparable to that of the wt until the ninth to tenth hour of growth. Thereafter, growth of the mutant strain practically ceased, thereby indicating exhaustion of amino acids as an alternative energy source that the bacteria had used instead of glucose due to the disturbed glycolytic pathway (Raghunathan et al., 2010). Accordingly, when we supplemented the medium with tryptone as a rich source of amino acids the growth of the *gapA* mutant was almost comparable with that of the wt strain (Figure 1B). In the *gapA*in mutant strain, the growth defect was even greater, thus indicating the polar effect of the insertion mutagenesis (Supplementary Figures 1, 2). The course of growth in brain heart infusion medium of wt and *gapA* mutant strains revealed the same trend as seen in the standard Chamberlain's medium (data not shown).

#### Ability of *gapA* mutant to invade and replicate within the host cells

**Figure 2:**
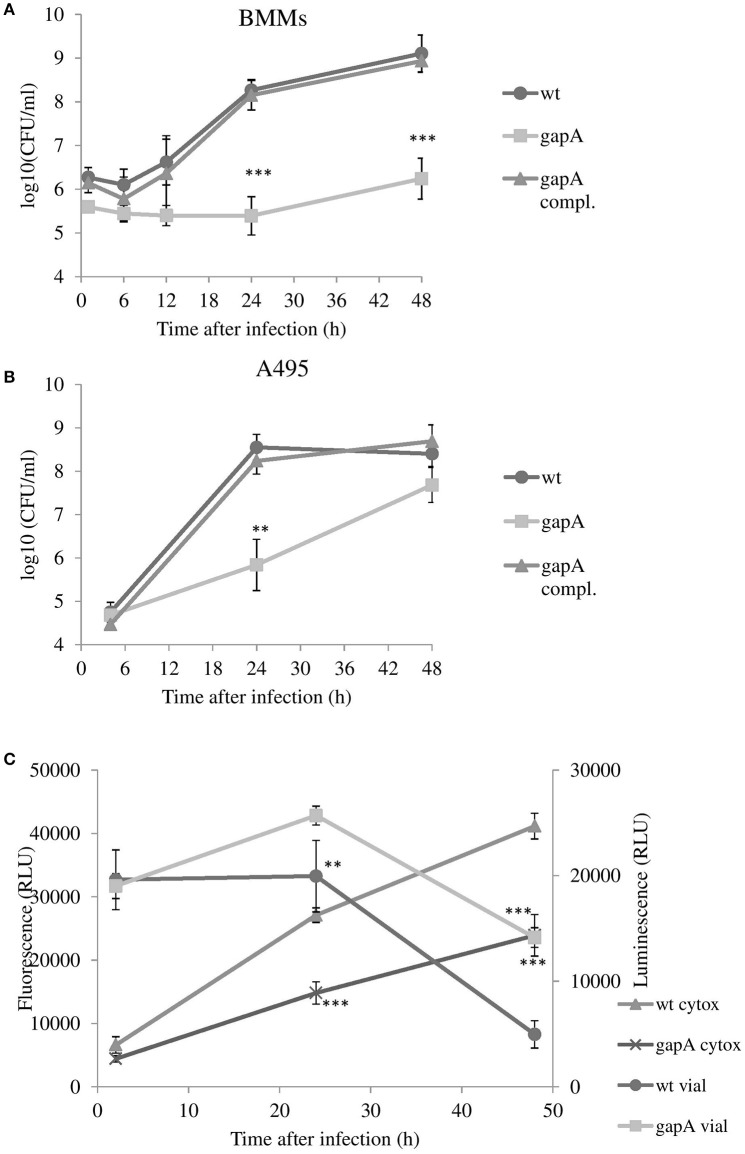
*In vitro* invasion and proliferation of *gapA, gapA*-complemented, and wt FSC200 in bone marrow macrophages (BMMs) **(A)** and A549 cells **(B)**. The cells were infected at MOI of 50:1 (BMM) or 200:1 (A549) with the indicated strains. BMMs were harvested at 1, 6, 12, 24, and 48 h and A549 at 4, 24, and 48 h post-infection. The numbers of bacteria recovered from the cells were counted as cfu. The data represent means ± *SD* of three independent experiments performed in triplicate. **(C)** Viability of cells and induction of cytotoxicity determined in BMMs infected with *gapA* mutant or wild-type FSC 200 strains. At 2, 24, and 48 h the cells were assayed using RealTime-Glo™ MT Cell Viability Assay kit and CellTox™ Green Cytotoxicity Assay kit (Promega). Data are means ± *SD* of triplicate samples and the results shown are representatives of three independent experiments. Asterisks indicate statistically significant differences; **P < 0.01; ***P < 0.001 (comparing *gapA* with the wild-type FSC200 strain).

Macrophages are believed to be the primary host cells for *F. tularensis in vivo*. To examine the ability of the *gapA* mutant to enter and replicate in macrophages, the BMMs were infected with the wt strain, the *gapA* mutant strain, and *gapA*-complemented strain at MOI 50:1 and numbers of intracellular bacteria were determined at 1, 6, 12, 24, and 48 h after infection (Figure 2A). Whereas, uptake of the *gapA* mutant seemed to be unaffected, the bacteria without the functional *gapA* gene revealed a significant defect in replication inside the BMMs. The numbers of bacteria did not change until 24 h post-infection. At that time, *gapA* bacteria started to replicate very slightly. Nevertheless, their intracellular amounts remained significantly reduced compared to those of the wt strain at 48 h. The same trend also was observed by immunofluorescence microscopy (data not shown).

*Francisella* has also been known to evade and replicate in non-phagocytic cells, such as epithelial cells. In a human alveolar epithelial cell line, the entry of *gapA* mutant bacteria also was comparable to that of the wt strain. The numbers of wt bacteria increased rapidly during the first 24 h and then stagnated until hour 48. In contrast, the *gapA* mutant strain replicated more slowly and showed a statistically significant growth defect at 24 h post-infection. The diminished growth continued to 48 h post-infection, at which time the number of microbes reached nearly the same level as that of the wild counterpart. The growth of bacteria was fully restored to wild-type levels by *gapA* complementation in both BMMs and A549, thereby demonstrating the contribution of the GapA protein to the ability of *F. tularensis* to replicate inside the host cell (Figure 2B).

We can conclude from this that the *gapA* mutant is able to invade and survive in both phagocytic and non-phagocytic cells. On the other hand, the *gapA* mutant exerts a significant replication defect that was far more pronounced in macrophages. However, we cannot exclude that this growth abolition can partly reflects the inability of the mutant strain to utilize glucose as main energy source and exhausted spare alternative intracellular sources of energy.

#### Effect of *gapA* deletion on *F. tularensis* cytotoxicity in BMMs

We also investigated the role of *gapA* gene deletion on host cell viability together with its cytopathogenic effects. We infected BMMs with the wt strain or the *gapA* mutant and monitored the cell viability and cytotoxicity simultaneously over the 48 h time period using the commercial kits RealTime-Glo™ MT Cell Viability Assay and CellTox™ Green Cytotoxicity Assay (Figure 2C). Both these kits can be multiplexed and enable the monitoring of both parameters in real time. The luminescent viability assay determines the number of viable cells in culture by measuring the reducing potential of cells and thus metabolism. The fluorescent cytotoxicity assay measures changes in membrane integrity that occur as a result of cell death. At 2 h post-infection, the luminescence and fluorescence values were nearly the same in both infected and non-infected groups of cells. At 24 h after infection, both *F. tularensis* strains revealed a cytotoxic effect on BMMs that deepened over the next 24 h (to 48 h post-infection). The effect of the *gapA* mutant, however, was significantly less pronounced in comparison to that of the wt strain. The same trend could be observed also in cell viability. Taken together the deletion of gapA gene resulted in a strain with significantly less detrimental effects on cell membrane integrity and viability compared to the parental strain.

#### Effect of *gapA* deletion on virulence attenuation and protection in the mouse infection model for tularemia

Groups of at least five BALB/c mice were infected subcutaneously using different doses of the *gapA* mutant (1.5 × 10^2^, 3 × 10^2^, 3 × 10^5^, and 3 × 10^7^) and then monitored for progression of the disease for 42 days (Table 4). The numbers of administered bacteria was confirmed by plating. The mice infected with wt FSC200 strain at doses 1.5 × 10^2^ and 3 × 10^2^ cfu died within 5–6 or 6–8 days, respectively. The *gapA* mutant, by contrast, showed a certain degree of attenuation as 60% of mice survived infection even with the highest dose of 3 × 10^7^ cfu/mouse. Unfortunately, we could not reveal any dose dependence between the challenge dose and rate of survival, because there was no significant difference between the percentage of mice surviving the highest and lower doses: 56.7% of mice in the challenge with 3 × 10^5^ cfu survived as did 66.7% of those infected with 3 × 10^2^ cfu. At the lowest dose of 1.5 × 10^2^ cfu, 76% of mice survived the infection. Complementation of the *gapA* mutant restored the virulence to the same levels as seen with the wt strain.

**Table 4:** Survival of mice infected with *gapA, gapA*-complemented, and wild-type strain and protective efficacy of *gapA* mutant strain.

**Strain**	**Inoculation dose (CFU)^a^**	**Time to death (day)^b^**	**% of survival^c^**	**wt FSC200 re-challenge dose (CFU)^d^**	**% of survival^e^**
wt FSC200	3 × 10^2^	5–6	0	–	–
	1.5 × 10^2^	6–8	0	–	–
*gapA* complemented	3 × 10^2^	5–6	0	–	–
	1.5 × 10^2^	6–9	0	–	–
*gapA* mutant	3 × 10^7^	5–11	60%	3 × 10^2^	100%
	3 × 10^5^	5–11	56.7%	3 × 10^2^	100%
	3 × 10^2^	5–11	66.7%	3 × 10^2^	100%
	1.5 × 10^2^	8–14	76%	3 × 10^2^	100%

a *BALB/c mice were subcutaneously infected with the indicated inoculum dose indicated as CFU/mouse*.

b *Time range in days when the mice died as consequence of infection*.

c *Percentage of animals surviving infection by indicted F. tularensis strain*.

d *Mice that survived the infection with inoculation dose were challenged with wild-type FSC200 strain on day 42 and monitored for signs of infection for 21 days*.

e *Percentage of animals immunized with different doses of gapA mutant that survived the rechallenge with wild-type strain FSC200*.

**Figure 3:**
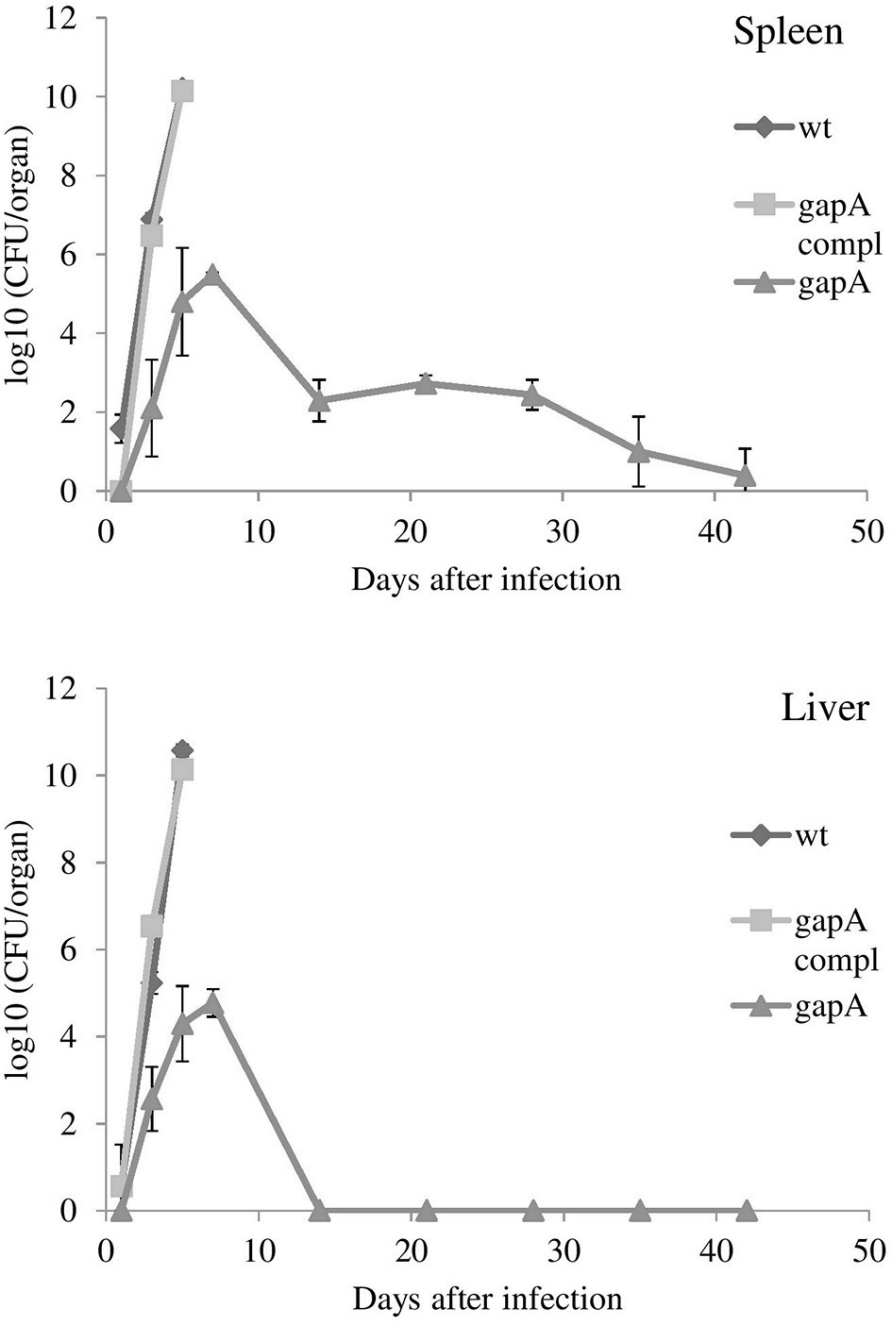
*In vivo* proliferation and dissemination of *gapA, gapA*-complemented, and wt FSC200 in spleen and liver of BALB/c mice inoculated s.c. with 3 × 10^2^ cfu/mouse of the indicated strains. Mice infected with the wt FCS200 strain or *gapA*-complemented strain died after day 5. Results are shown as the average log_10_ cfu per organ ± *SD* at the indicated time point of infection. The results are representative of three independent experiments.

Those mice that survived administration of the *gapA* mutant strain were rechallenged s.c. with the full virulent wt FSC200 strain at day 42. All mice immunized with the four different doses of the *gapA* mutant survived the wt challenge and displayed no symptoms of tularemia disease during the following 3 weeks. Next, we evaluated the ability of the *gapA* mutant to disseminate through and persist within host tissues after s.c. infection. Groups of at least three BALB/c mice were inoculated subcutaneously with a dose of 3 × 10^2^ cfu/mouse. The bacterial burdens were determined in the spleen and liver tissue homogenates at days 1, 3, 5, 7, 14, 28, 35, and 42 after infection (Figure 3). The numbers of wt and complemented strains increased rapidly in both organs from the first day and reached almost 10^10^ cfu in both organs. Mice infected with those two strains did not survive beyond day 5. On the other hand, the *gapA* mutant could be detected in all organs only from day 3. Thereafter, the mutant bacteria replicated quite rapidly, reached the maximal burdens of ~10^5^ cfu in the spleen and liver, then declined precipitously. By day 14, the bacteria had been completely eliminated from the liver. In the spleen, however, the initial quick decline ceased at day 14 and the bacteria persisted there at approximately the same levels of 10^2^ cfu/spleen. After day 28 post-infection, those levels continued to decrease slowly. At day 42, the bacteria could still be detected in spleens of some inoculated mice in small amounts (<75 cfu). These data indicated that the *gapA* mutant is able to infect mice and to persist in infected organs, but its ability to replicate inside the host tissues is disrupted.

### Localization of *F. Tularensis gapA* protein

**Figure 4:**
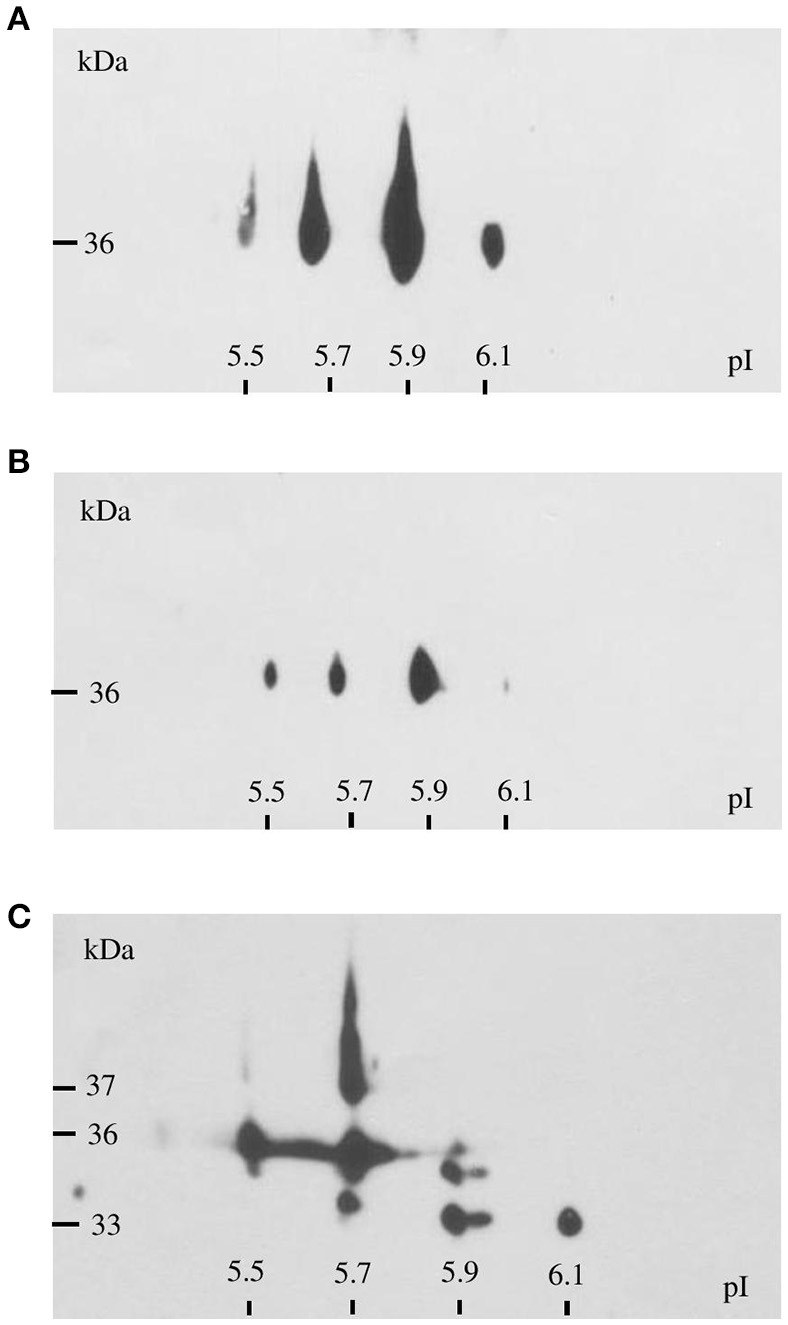
Immunodetection of GapA protein in *F. tularensis* FSC200 whole-cell lysate **(A)**, crude membrane fraction **(B)**, and culture filtrate proteins **(C)** following 2D SDS-PAGE separation with separation in non-linear pH range 3–10 in the first dimension followed by separation on gradient 9–16% SDS-PAGE gel in the second dimension.

GAPDH is encoded in *F. tularensis* by a single gene, *gapA*. According to the PSORT-B program (http://www.psort.org/psortb/), GapA is predicted to be a cytosolic protein. There is increasing evidence, however, of its extracytosolic localizations in other bacteria, often in connection with virulence. We thus decided to explore the localization of GapA in *F. tularensis* using various techniques. First, we analyzed whole-cell lysates, fractions enriched in membrane proteins, and CFPs representing the secreted proteins of *F. tularensis* by two-dimensional gel electrophoreses followed by western blot and immunodetection of GapA protein with polyclonal antibody. Figure 4 shows that GapA occurred in at least four charge variants in all tested fractions. Particularly noteworthy is that the immunodetection of GapA in CFPs revealed mass variants not detected in whole-cell lysates or membrane fractions. These data show for the first time the presence of GapA in other compartments besides the cytosol, post-translational modifications of this protein, and changes in the protein size that might be associated with the secretion process.

**Figure 5:**
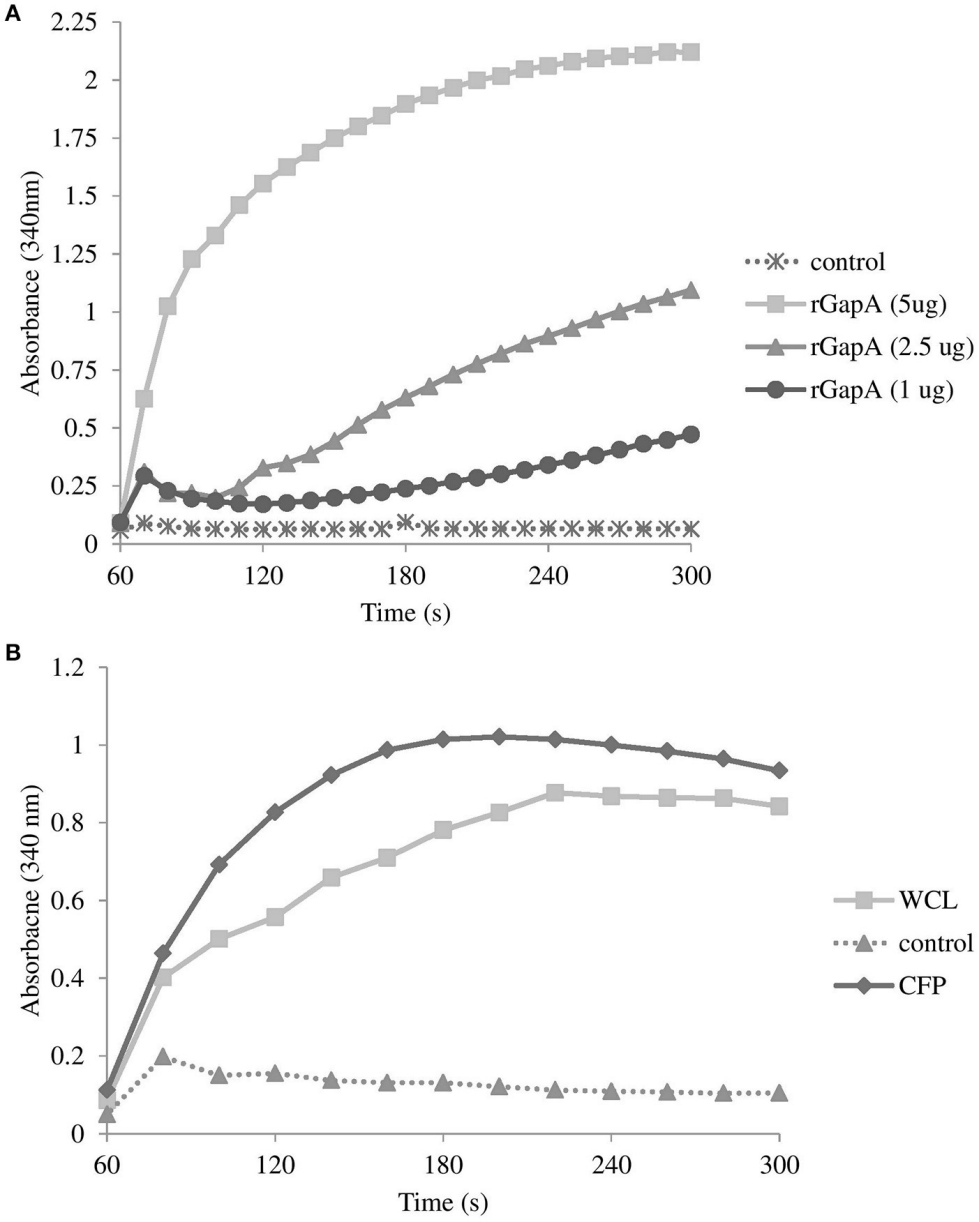
Glyceraldehyde-3-phosphate dehydrogenase activity associated with purified recombinant GapA at concentrations 5, 2.5, and 1 μg **(A)**; whole-cell lysate and culture filtrate proteins (both 50 μg) from *F. tularensis* FSC200 **(B)** determined by the conversion of NAD^+^ to NADH as described in the section Materials and Methods. Results shown from one experiment are representatives of two independent experiments.

Further evidence of the GapA extracellular localization was obtained by the analysis of its catalytic activity. As seen in Figure 5A, the purified recombinant protein GapA is able to catalyze the NADH formation in the presence of the substrate glyceraldehyde-3-phosphate. As expected, the whole-cell lysates reported the GAPDH activity, as well. Furthermore, the CFPs also showed significant NADH formation coupled to glyceraldehyde-3-phosphate oxidation (Figure 5B). Unfortunately, due to problems with the solubility in reaction buffer, we were not able to demonstrate the catalytic activity of the fraction enriched in membrane proteins.

**Figure 6:**
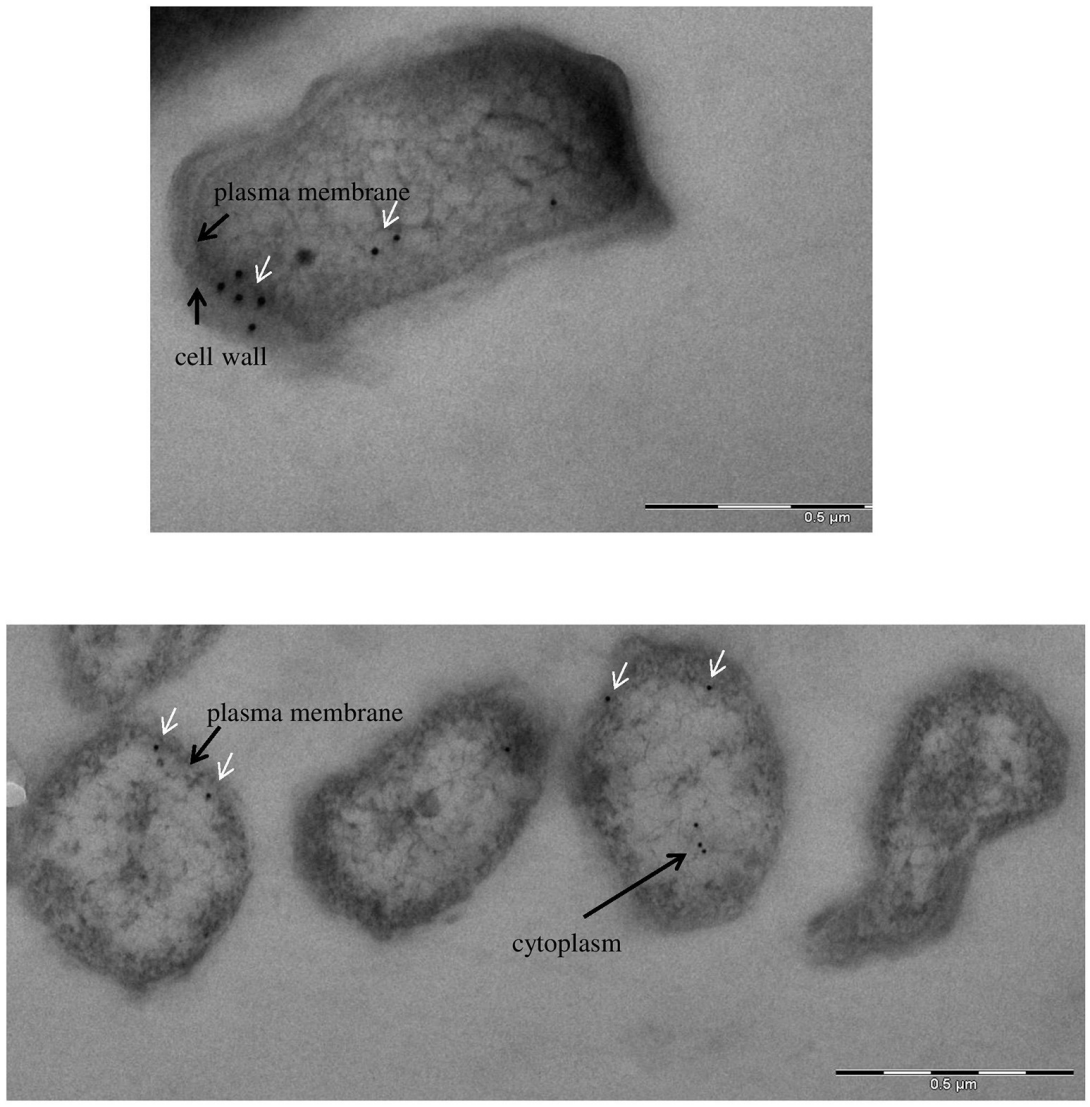
Immunoelectron microscopy detection of glyceraldehyde-3-phosphate dehydrogenase: subcellular distribution of GapA in *F. tularensis* FSC200 grown in Chamberlain's medium. Cell cultures were fixed and processed as described in the section Materials and Methods. The GapA protein (white arrows) is detected in the bacteria's cytoplasm, plasma membrane, and cell wall.

To define more precisely the cellular localization of GapA, we used *F. tularensis* FSC200 grown in Chamberlain's medium and processed for transmission electron microscopy. The immunolabeling experiments were performed on ultrathin sections as described in the section Materials and Methods. Electron microscopy revealed the presence of GapA protein mainly in the cytoplasm, which is consistent with its intracellular glycolytic function, and in the plasma membrane. In some cases, the protein appears in the cell wall, which might reflect the GapA trafficking to extracellular space (Figure 6).

### Binding of recombinant GapA protein to host proteins

**Figure 7:**
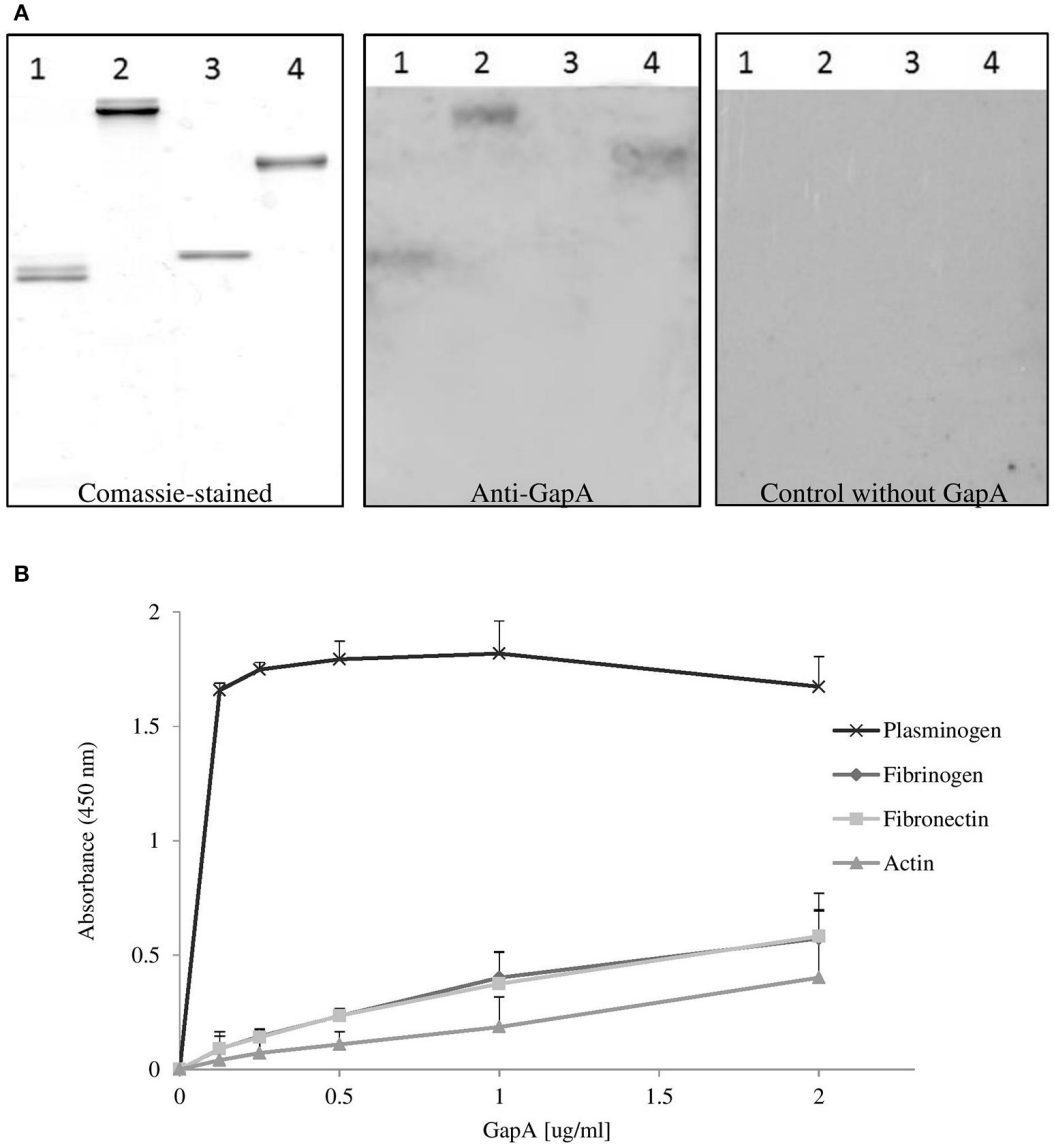
Binding of *F. tularensis* GapA to selected host proteins. **(A)** Far-western analysis of GapA binding to PVDF-immobilized human proteins. The human proteins fibrinogen (lane 1), fibronectin (lane 2), actin (lane 3), plasminogen (lane 4) were separated on SDS-PAGE and the gel was either Coomassie blue-stained (first panel) or electroblotted. After the PVDF membrane had reacted with the recombinant GapA protein, it was incubated with anti-GapA antibodies and processed to visualize reactive bands (second panel). The third panel shows detection when the incubation with GapA was omitted. **(B)** Solid-phase binding assay of human proteins (plasminogen, fibrinogen, fibronectin, and actin) coated on 96-well microtiter plate and reacted with different concentrations of GapA. Data are presented as means ± *SD* from three independent experiments.

Binding studies were performed to investigate the possible interaction of purified recombinant *F. tularensis* GapA with selected human proteins (plasminogen, fibrinogen, fibronectin, actin) known to interact with the GAPDH of other pathogens (summarized in Giménez et al., 2014). The results from far-western blot and solid-phase ligand-binding assays showed that purified GapA is able to bind to serum proteins plasminogen, fibrinogen, and fibronectin (Figures 7A,B). On the other hand interaction with cytoskeletal protein actin could not be proved this way.

## Discussion

In general, the bacterial DsbA proteins catalyze the disulfide bond formation in a wide array of virulence factors essential for pathogenesis of bacteria, thus putting them at the center of interest as potential targets for the development of new therapeutic and prophylactic agents (Smith et al., 2016). During the past decade, several working groups, including our own, have studied intensively the DsbA protein of *F. tularensis* in its many aspects. The data have been presented in a number of publications (summarized by Rowe and Huntley, 2015). On the whole, FtDsbA ensures the correct folding of extracytosolic proteins through its oxidoreductase, isomerase, and probably also chaperone activities. The inactivation of the *dsbA* gene had a pleiotropic effect, resulting from the accumulation of a number of misfolded and thus dysfunctional and unstable proteins. This ultimately is reflected in reduced virulence in mice, as well as defects in intracellular survival and phagosomal escape. The two most recently published studies were directed to trapping and identification of FtDsbA substrates in order to detect new *F. tularensis* virulence factors (Ren et al., 2014); (Qin et al., 2016). In contrast to those studies, we provide here deeper insights into the complex proteome changes induced by the *dsbA* gene deletion. Using SILAC metabolic labeling, we were able to identify more than 60 proteins whose levels in the bacterial cells might be influenced directly or indirectly by the DsbA protein. The overlap of our analysis with both the trapping studies is quite limited, thus indicating far more extensive effect of the DsbA protein on other proteins and cellular processes mediated indirectly through the substrates. Only three proteins (hypothetical proteins FTS_1749 and FTS_1279 plus D-alanyl-D-alanine carboxypeptidase) were identified in common across the three studies. It should be noted, however, that even the two trapping studies overlapped in only 25 proteins. For example, Qin et al. (2016) presumed from their results that several components of the FPI participating in the type VI secretion system (IglC, IglB, and PdpB) might be substrates of DsbA protein in subsp. *tularensis* (referred to here as FipB). This observation was not confirmed, however, by Ren et al. (2014). In our study, we detected significant elevation in only one FPI component (IglE), even though two of the aforementioned proteins (IglC and IglB) are quite abundant and detected reliably in the *Francisella* membranome (Pávková et al., 2005). One of the hypothetical proteins, FTS_0450, found to be elevated in the *dsbA* mutant is identical with FevR and might provide indirect evidence for changes in the secretion system. This transcriptional regulator is required for the expression of genes in the MglA/SspA regulon including all the *Francisella* pathogenicity island genes. Hence, the change of the FevR level can cause dysregulation in the FPI genes transcription, including in components of the type VI secretion system (Brotcke and Monack, 2008; Bröms et al., 2010).

In order to identify new candidates responsible for the *dsbA* mutant strain attenuation we inactivated 15 genes encoding proteins whose DsbA dependent regulation has not been observed yet. Based on selection criteria we further focused on gene encoding cytosolic glycolytic enzyme, glyceraldehyde-3-phosphate dehydrogenase, whose level was significantly elevated in the *dsbA* mutant strain. This protein was previously shown to be immunoreactive, (Havlasová et al., 2005; Chandler et al., 2015; Straskova et al., 2015), and identified by proteomic analyses in fractions of enriched membrane proteins (Pávková et al., 2005) and CFPs (Konecna et al., 2010). These previous findings were in accordance with an increasing number of studies demonstrating additional activities of GAPDH not related to its primary metabolic function in both eukaryotic and prokaryotic cells. Many reports describe extracellular localization of GAPDH in Gram-positive (*Streptococcus* ssp., *Mycoplasma pneumoniae, Listeria monocytogenes, Bacillus anthracis*) and several Gram-negative (*E. coli, Neisseria meningitidis, Brucella abortus, Edwardsiella tarda*) bacteria suggesting that this protein possesses other roles in addition to its glycolytic function including the involvement in host-pathogen interactions (summarized by Henderson and Martin, 2011; Giménez et al., 2014). Nevertheless, the gene encoding GAPDH is essential for survival of many bacteria, which limits the analysis of its role in pathogenic and virulence processes.

In *Francisella* ssp., GAPDH is encoded by a single gene (*gapA*) and is highly conserved within the subspecies. As in other bacteria, its amino acid sequence shows no predicted signal sequence, membrane-spanning motif, or functional domain other than GAPDH NAD binding domain. Our experimental data show that GapA of *F. tularensis* subsp. *holarctica* FSC200 strain is an active enzyme able to catalyze the oxidative phosphorylation of glyceraldehyde-3-phosphate to 1,3-diphosphoglycerate in the presence of inorganic phosphate and NAD+, the sixth step of glycolysis. GapA had previously been regarded as non-essential for *Francisella* (Meibom and Charbit, 2010; Raghunathan et al., 2010). Here, we provide experimental evidence for this prediction inasmuch as the in-frame deletion of *gapA* resulted in a viable mutant strain with specific phenotype. The observed *gapA*-related phenotype was fully complemented in the *gapA*-complemented strain, indicating no polar effect of *gapA* gene deletion on surrounding genes. The glycolytic pathway is known to be complete in *F. tularensis*, but it is not critical for its intracellular survival and virulence inasmuch as the bacteria seem to prefer utilizing specific amino acids for energy production during the infection process (Raghunathan et al., 2010; Brissac et al., 2015). However, the *gapA* mutant revealed an obvious extracellular growth defect as it was able to grow in standard chemically defined Chamberlain's medium to the same extent as does the wild-type strain only until exhaustion of the amino acids as alternative source of energy.

In several pathogenic bacteria like *Neisseria meningitides* (Tunio et al., 2010) and *Streptococcus pyogenes* (Jin et al., 2005), the GAPDH was found to be required for optimal adhesion and subsequent invasion to host cells. This is not the case for the *F. tularensis* GapA as the entry of the *gapA* mutant into the cells was comparable to the parental strain in both the epithelial cell line and primary macrophages. The reduced bacterial numbers recovered from infected cells in the late stages of infection (24 h and later) can be associated with the disturbed glycolytic pathway (author note: the phagosomal escape in BMM's was not affected so far—data not shown). The consequence of this metabolic defect can also contribute to the attenuated phenotype of the *gapA* mutant in the mouse model of infection.

The alternative functions of metabolic enzymes in pathogenic bacteria are related to their cell surface display and/or secretion. This localization enables the proteins to bind to host components and thus to participate in pathogenic processes. GAPDH has been found on the cell surface or even secreted in many Gram-positive (streptococci and staphylococci) and Gram-negative (e.g., *E. coli, N. meningitidis*, and *E. tarda*) bacteria, thereby facilitating the colonization and manipulation of host cells (summarized by Giménez et al., 2014). The relationship between extracellular localization of GAPDH and virulence has been revealed in several published studies. For example, only the pathogenic *E. coli* strains were able to secrete GAPDH into the culture medium (Egea et al., 2007). A mutant strain of *S. pyogenes* unable to export GAPDH from the cytosol to the cell surface revealed significant attenuation for virulence in the mouse model (Jin et al., 2011). Our previous proteomic analysis indicated extracellular localization of *F. tularensis* GapA, and in this study we verified this finding using several different approaches. Our data show that GapA occurs in various compartments involving the cytosol, cell wall, and extracellular medium. The possibility of cytosolic contamination of the fraction enriched in CFPs had previously been ruled out by exploring the lactate dehydrogenase activity (Konecna et al., 2010). On 2-D gels, the GapA could be detected in multiple spots that differ in isoelectric points. Several additional mass variants were found on 2-D gels with separated CFPs. The presence of multiple GAPDH variants on 2-D gels attributable to post-translational modifications has been widely reported in other microorganisms (Henderson and Martin, 2011; Giménez et al., 2014). The surface-localized or extracellularly secreted GAPDH of pathogenic bacteria is in general known to bind to a number of host proteins The most commonly described binding of bacterial GAPDH to human plasminogen, fibrinogen, or fibronectin plays a role in degradation of extracellular matrix proteins that facilitate bacterial dissemination within the host organism (summarized by Giménez et al., 2014). As *F. tularensis* is an intracellular pathogen we wanted to test whether its GAPDH homolog (GapA) has retained the ability to bind to those serum proteins. Using two *in vitro* systems, we could demonstrate the binding of GapA to selected host plasma components with strong preference for plasminogen compared to fibrinogen and fibronectin. However, further studies are needed to demonstrate the real meaning of these binding activities.

In conclusion, this extensive analysis of the *F. tularensis* subsp. *holarctica* membrane proteome enabled the discovery of a number of proteins that are directly or indirectly affected by the functional DsbA protein. These proteins are involved in various cellular processes and many of them might significantly contribute to the pleiotropic phenotype of *dsbA* mutant strain described in several previously published studies. In this study, we selected GADPH of *F. tularensis* for further investigation as this protein is known to be multifunctional in other pathogenic bacteria and there is a growing evidence for its role in host-pathogen interaction. In this study we analyzed the basic features of *Francisella* GAPDH that are common for other bacterial homologs and indicate its multifunctional character. Accordingly we demonstrated the extracytosolic localization of *Francisella* GAPDH and its ability to bind several host serum proteins. Nevertheless it seems to be worth to perform further analysis on this protein. Next studies are underway to elucidate the real role of the secreted GAPDH of *F. tularensis*. They include the experimental manifestation of GapA secretion GapA into the cytoplasm of host cells and the detection and identification of potential intracellular interaction partners.

## Author contributions

IP, JK, and JS designed the experiments; IP, JK, MK, MoS, VS, MaS, and JZ performed the experiments; IP, JK, MaS, and MK analyzed samples; IP, JK, MaS, PH, and JS interpreted the data; and IP, JK, and JS wrote the manuscript.

### Conflict of interest statement

The authors declare that the research was conducted in the absence of any commercial or financial relationships that could be construed as a potential conflict of interest.
