# Supplementary material for: The Multiple Localized Glyceraldehyde-3-Phosphate Dehydrogenase Contributes to the Attenuation of the Francisella tularensis dsbA Deletion Mutant
Source: Front Cell Infect Microbiol. 2017 Dec 11;7:503. doi: 10.3389/fcimb.2017.00503 (PMC5732180; doi:10.3389/fcimb.2017.00503)
Supplement: Supplementary file 1 [file Table1.DOCX]

**SUPPLEMENTARY TABLE 1 |** Plasmids used for TargeTron insertional mutagenesis.

| Protein name | FTS_locus tag | Primer | 5´- 3´ sequence |
| --- | --- | --- | --- |
| Hypothetical protein | FTS_1749 | IBS | AAAACTCGAGATAATTATCCTTAGCAAACTTAACGGTGCGCCCAGATAGGGTG |
|  |  | EBS1d | CAGATTGTACAAATGTGGTGATAACAGATAAGTCTTAACGGATAACTTACCTTTCTTTGT |
|  |  | EBS2 | TGAACGCAAGTTTCTAATTTCGATTTTTGCTCGATAGAGGAAAGTGTCT |
|  |  | F | TGGAAATGAATGAATCTGGCG |
|  |  | R | GCTCGCCATCCTTATCTTTG |
| Hypothetical protein | FTS_0495 | IBS | AAAACTCGAGATAATTATCCTTAGTAGTCATATGCGTGCGCCCAGATAGGGTG |
|  |  | EBS1d | CAGATTGTACAAATGTGGTGATAACAGATAAGTCATATGCCTTAACTTACCTTTCTTTGT |
|  |  | EBS2 | TGAACGCAAGTTTCTAATTTCGATTACTACTCGATAGAGGAAAGTGTCT |
|  |  | F | CATTTTGTGGCGATAACTGTAG |
|  |  | R | TCATACAACCTGCTACATCAATA |
| Glycerophosphoryl diester phosphodiesterase | FTS_1476 | IBS | AAAACTCGAGATAATTATCCTTACTATTCCACCAGGTGCGCCCAGATAGGGTG |
|  |  | EBS1d | CAGATTGTACAAATGTGGTGATAACAGATAAGTCCACCAGAATAACTTACCTTTCTTTGT |
|  |  | EBS2 | TGAACGCAAGTTTCTAATTTCGGTTAATAGTCGATAGAGGAAAGTGTCT |
|  |  | F | CAACTGCTCTAACTGTCTGTA |
|  |  | R | TAAAGCCTGCCATTCAAAAGAT |
| Hypothetical protein | FTS_0402 | IBS | AAAACTCGAGATAATTATCCTTACAACACGCAGTTGTGCGCCCAGATAGGGTG |
|  |  | EBS1d | CAGATTGTACAAATGTGGTGATAACAGATAAGTCGCAGTTAATAACTTACCTTTCTTTGT |
|  |  | EBS2 | TGAACGCAAGTTTCTAATTTCGGTTTGTTGTCGATAGAGGAAAGTGTCT |
|  |  | F | AACTTCTTTTATAATCATCTCCTT |
|  |  | R | GTCATCATCATCTATTTGCTCA |
| Glyceraldehyde-3-phosphate dehydrogenase/erythrose-4-phosphate dehydrogenase | FTS_1117 | IBS | AAAACTCGAGATAATTATCCTTACATCTCTTTCAGGTGCGCCCAGATAGGGTG |
|  |  | EBS1d | CAGATTGTACAAATGTGGTGATAACAGATAAGTCTTTCAGCATAACTTACCTTTCTTTGT |
|  |  | EBS2 | TGAACGCAAGTTTCTAATTTCGGTTAGATGTCGATAGAGGAAAGTGTCT |
|  |  | F | CTGCTCAAGGTAGATTCTCAA |
|  |  | R | GCGGCACCAGTTGAGTTAG |
| Hypothetical protein | FTS_0659 | IBS | AAAACTCGAGATAATTATCCTTAAGTGTCGCCGTAGTGCGCCCAGATAGGGTG |
|  |  | EBS1d | CAGATTGTACAAATGTGGTGATAACAGATAAGTCGCCGTACATAACTTACCTTTCTTTGT |
|  |  | EBS2 | TGAACGCAAGTTTCTAATTTCGATTACACTTCGATAGAGGAAAGTGTCT |
|  |  | F | GTCAATAGTAATGGTAATGTCG |
|  |  | R | ATAGGCGTTTGCTTGTAATCTT |
| GTP-dependent nucleic acid-binding protein EngD | FTS_0935 | _IBS | AAAACTCGAGATAATTATCCTTAAGTAACGGCGAAGTGCGCCCAGATAGGGTG |
|  |  | EBS1d | CAGATTGTACAAATGTGGTGATAACAGATAAGTCGGCGAAGGTAACTTACCTTTCTTTGT |
|  |  | EBS2 | TGAACGCAAGTTTCTAATTTCGATTTTACTTCGATAGAGGAAAGTGTCT |
|  |  | F | ATGGGATTTAAATGTGGTATAGT |
|  |  | R | GGCACCTTTTTCACCATTATAT |
| Hypothetical protein | FTS_0974 | IBS | AAAACTCGAGATAATTATCCTTATTTTACTACAAAGTGCGCCCAGATAGGGTG |
|  |  | EBS1d | CAGATTGTACAAATGTGGTGATAACAGATAAGTCTACAAAAGTAACTTACCTTTCTTTGT |
|  |  | EBS2 | TGAACGCAAGTTTCTAATTTCGGTTTAAAATCGATAGAGGAAAGTGTCT |
|  |  | F | ACTTTGTTTTCTTGTCTAGGCT |
|  |  | R | ATCTAAAACAAAACCATCAACGA |
| Acyltransferase | FTS_0078 | IBS | AAAACTCGAGATAATTATCCTTAGCCTACGCGTACGTGCGCCCAGATAGGGTG |
|  |  | EBS1d | CAGATTGTACAAATGTGGTGATAACAGATAAGTCGCGTACTTTAACTTACCTTTCTTTGT |
|  |  | EBS2 | TGAACGCAAGTTTCTAATTTCGATTTAGGCTCGATAGAGGAAAGTGTCT |
|  |  | F | CAAGAATGCAAGTTTTTAAGGTA |
|  |  | R | ACACATATCTTTTAATTCAGCGA |
| Acyltransferase | FTS_0079 | IBS | AAAACTCGAGATAATTATCCTTAGACAGCATTTCAGTGCGCCCAGATAGGGTG |
|  |  | EBS1d | CAGATTGTACAAATGTGGTGATAACAGATAAGTCATTTCAGCTAACTTACCTTTCTTTGT |
|  |  | EBS2 | TGAACGCAAGTTTCTAATTTCGATTCTGTCTCGATAGAGGAAAGTGTCT |
|  |  | F | TTTGGAATATTTTAATGTGGT |
|  |  | R | ACCAAAATCCATCCTAGCTATA |
| Hypothetical protein | FTS_1229 | IBS | AAAACTCGAGATAATTATCCTTAACTACCACTGAAGTGCGCCCAGATAGGGTG |
|  |  | EBS1d | CAGATTGTACAAATGTGGTGATAACAGATAAGTCACTGAACATAACTTACCTTTCTTTGT |
|  |  | EBS2 | TGAACGCAAGTTTCTAATTTCGATTGTAGTTCGATAGAGGAAAGTGTCT |
|  |  | F | CTAAATACATCAAACGCAAAAGA |
|  |  | R | CGAGAGCCATTTTTCATCAAC |
| Hypothetical protein | FTS_0920 | IBS | AAAACTCGAGATAATTATCCTTAGGTAACACCAGAGTGCGCCCAGATAGGGTG |
|  |  | EBS1d | CAGATTGTACAAATGTGGTGATAACAGATAAGTCACCAGAGATAACTTACCTTTCTTTGT |
|  |  | EBS2 | TGAACGCAAGTTTCTAATTTCGATTTTACCTCGATAGAGGAAAGTGTCT |
|  |  | F | GATTCACATATTCATTTCTGGG |
|  |  | R | TCTTTACATTTGACATTTGATTATT |
| Hypothetical protein | FTS_0676 | IBS | AAAACTCGAGATAATTATCCTTATAAAACATGAGTGTGCGCCCAGATAGGGTG |
|  |  | EBS1d | CAGATTGTACAAATGTGGTGATAACAGATAAGTCATGAGTTGTAACTTACCTTTCTTTGT |
|  |  | EBS2 | TGAACGCAAGTTTCTAATTTCGGTTTTTTATCGATAGAGGAAAGTGTCT |
|  |  | F | GCAACGGCAATGTAGCAGC |
|  |  | R | AAACATCATAGAAATCATCACCA |
| Sugar porter (SP) family protein | FTS_0580 | IBS | AAAACTCGAGATAATTATCCTTATTGATCTAGGTGGTGCGCCCAGATAGGGTG |
|  |  | EBS1d | CAGATTGTACAAATGTGGTGATAACAGATAAGTCTAGGTGTGTAACTTACCTTTCTTTGT |
|  |  | EBS2 | TGAACGCAAGTTTCTAATTTCGGTTATCAATCGATAGAGGAAAGTGTCT |
|  |  | F | AGGTGTTTGCTCTGGGTTTG |
|  |  | R | GTTCTTTATTTGTCATTTCTGGT |
| Excinuclease ABC, subunit A | FTS_1439 | _IBS | AAAACTCGAGATAATTATCCTTAATACCCCGGGATGTGCGCCCAGATAGGGTG |
|  |  | EBS1d | CAGATTGTACAAATGTGGTGATAACAGATAAGTCCGGGATAATAACTTACCTTTCTTTGT |
|  |  | EBS2 | TGAACGCAAGTTTCTAATTTCGATTGGTATTCGATAGAGGAAAGTGTCT |
|  |  | F | CGCAAAAACCCATAACCTAAAA |
|  |  | R | GCCCTCCTTTACTACCACC |
| Hypothetical protein | FTS_0367 | IBS | AAAACTCGAGATAATTATCCTTATATTTCGCTATTGTGCGCCCAGATAGGGTG |
|  |  | EBS1d | CAGATTGTACAAATGTGGTGATAACAGATAAGTCGCTATTATTAACTTACCTTTCTTTGT |
|  |  | EBS2 | TGAACGCAAGTTTCTAATTTCGGTTAAATATCGATAGAGGAAAGTGTCT |
|  |  | F | TGAACACAATAATGACAAATACC |
|  |  | R | CGACCAGACAAAACTACTAATA |
