# Supplementary material for: The Multiple Localized Glyceraldehyde-3-Phosphate Dehydrogenase Contributes to the Attenuation of the Francisella tularensis dsbA Deletion Mutant
Source: Front Cell Infect Microbiol. 2017 Dec 11;7:503. doi: 10.3389/fcimb.2017.00503 (PMC5732180; doi:10.3389/fcimb.2017.00503)
Supplement: Supplementary file 2 [file Table2.DOCX]

**SUPPLEMENTARY TABLE 2 |** Protein identification and quantification results from the SILAC-based quantitative proteomic analysis.

| Accession | | Protein name | Σ# Unique Peptides | L/H | p-value |
| --- | --- | --- | --- | --- | --- |
| K0E4X6 | Uncharacterized protein, FTS_1495 | | 5 | 37,37 | 0,003 |
| K0E9A2 | Uncharacterized protein FTS_1538 | | 9 | 14,50 | 0,011 |
| K0E945 | Chitinase family 18 protein | | 24 | 14,48 | 0,007 |
| K0E9T0 | Uncharacterized protein FTS_1749 | | 25 | 10,18 | 0,009 |
| K0E1Q5 | Pyruvate phosphate dikinase | | 26 | 6,22 | 0,009 |
| K0E6Y1 | D-alanyl-D-alanine carboxypeptidase | | 23 | 5,74 | 0,017 |
| K0E5M6 | Uncharacterized protein FTS_0495 | | 9 | 5,11 | 0,004 |
| K0E7E4 | Uncharacterized protein FTS_1279 | | 18 | 4,95 | 0,028 |
| K0EAL7 | Siderophore biosynthesis protein | | 27 | 3,22 | 0,031 |
| K0E4V9 | Glycerophosphoryl diester phosphodiesterase | | 15 | 2,36 | 0,010 |
| K0E5H6 | Uncharacterized protein FTS_0402 | | 11 | 2,31 | 0,024 |
| K0E8B7 | Glyceraldehyde-3-phosphate dehydrogenase/erythrose-4-phosphate dehydrogenase | | 8 | 2,26 | 0,013 |
| K0EC19 | Catalase-peroxidase | | 27 | 2,24 | 0,013 |
| K0E7K7 | Parvulin-like peptidyl-prolyl isomerase domain-containing protein | | 7 | 2,13 | 0,005 |
| K0E3E9 | Uncharacterized protein FTS_0815 | | 5 | 2,11 | 0,043 |
| K0E5J5 | Uncharacterized protein FTS_0450 | | 3 | 2,10 | 0,012 |
| K0E355 | FAD binding family protein | | 10 | 2,08 | 0,017 |
| K0E7W0 | X-prolyl aminopeptidase 2 | | 21 | 1,91 | 0,045 |
| K0EAH4 | Elongation factor Tu | | 27 | 1,80 | 0,030 |
| K0E5Z0 | Uncharacterized protein FTS_0659 | | 15 | 1,75 | 0,014 |
| K0E7B1 | Uncharacterized protein FTS_1229 | | 6 | 1,62 | 0,037 |
| K0E632 | Uncharacterized protein FTS_0111/1139 | | 9 | 1,53 | 0,041 |
| K0E6R4 | von Willebrand factor type A domain-containing protein | | 5 | 0,66 | 0,023 |
| K0E3M5 | GTPase HflX | | 11 | 0,66 | 0,040 |
| K0E797 | Type IV pili, pilus assembly protein | | 1 | 0,65 | 0,040 |
| K0E8K7 | ATP synthase gamma chain | | 17 | 0,65 | 0,004 |
| K0E1V1 | LPS fatty acid acyltransferase | | 13 | 0,64 | 0,030 |
| K0E4N5 | Acyltransferase | | 10 | 0,63 | 0,013 |
| K0E8D4 | Ribonuclease HII | | 8 | 0,61 | 0,032 |
| K0E8W3 | ABC transporter ATP-binding protein | | 9 | 0,61 | 0,021 |
| K0E8K5 | Uncharacterized protein FTS_0137 | | 2 | 0,59 | 0,049 |
| K0ECP6 | ATP synthase subunit beta | | 32 | 0,59 | 0,005 |
| K0ECQ1 | F0F1 ATP synthase subunit C | | 1 | 0,59 | 0,008 |
| K0E9F9 | Drug:H+ antiporter-1 (DHA1) family protein | | 3 | 0,59 | 0,012 |
| K0EBZ1 | UvrABC system protein A | | 35 | 0,58 | 0,035 |
| K0E1Y3 | Uncharacterized protein FTS_0202 | | 10 | 0,58 | 0,031 |
| K0EAC6 | Nucleoside permease NUP family protein | | 5 | 0,57 | 0,026 |
| K0E2X8 | O-antigen flippase | | 6 | 0,56 | 0,042 |
| K0E9T3 | ATP synthase subunit delta | | 11 | 0,56 | 0,001 |
| K0EAJ2 | ATP synthase epsilon chain | | 10 | 0,55 | 0,004 |
| K0E5M3 | ATP-binding cassette (ABC) superfamily protein | | 5 | 0,52 | 0,000 |
| K0E959 | ATP-dependent RNA helicase | | 11 | 0,52 | 0,019 |
| K0E5D9 | ATP synthase subunit alpha | | 29 | 0,51 | 0,003 |
| K0E7Q3 | Amino acid transporter | | 4 | 0,51 | 0,007 |
| K0E8S0 | Uncharacterized protein FTS_0200 | | 9 | 0,49 | 0,009 |
| K0EB28 | Uncharacterized protein FTS_1068 | | 3 | 0,40 | 0,011 |
| K0E894 | Regulatory protein RecX | | 2 | 0,37 | 0,045 |
| K0E5T3 | Sugar porter (SP) family protein | | 4 | 0,33 | 0,002 |
| K0E8V2 | Glycosyl transferase family protein | | 5 | 0,31 | 0,024 |
|  |  | |  |  |  |
| K0E8B8 | Isochorismatase hydrolase family protein | | 7 | 4,91 | 0,005 |
| K0E783 | Uncharacterized protein FTS_1187 | | 8 | 3,84 | 0,010 |
| K0EAS2 | Uncharacterized protein FTS_0920 | | 6 | 3,32 | 0,001 |
| K0E9Y1 | Uncharacterized protein FTS_0676 | | 8 | 1,98 | 0,016 |
| K0E3Z0 | Dual-specificity RNA methyltransferase RlmN | | 4 | 0,66 | 0,034 |
| K0E6S3 | Uncharacterized protein FTS_0367 | | 3 | 0,63 | 0,020 |
| K0E2X3 | Membrane protein/O-antigen protein | | 1 | 0,60 | 0,027 |
| K0E526 | Delta-aminolevulinic acid dehydratase | | 8 | 0,53 | 0,006 |
| K0E7I4 | Uracil-DNA glycosylase | | 3 | 0,33 | 0,037 |
| K0E4N6 | Glycosyl transferases group 1 family protein | | 6 | 0,33 | 0,020 |
|  |  | |  |  |  |
| K0E6U8 | Uncharacterized protein FTS_0974 | | 6 | 36,12 | 0,036 |
| K0EAU8 | GTP-dependent nucleic acid-binding protein EngD | | 3 | 1,60 | 0,048 |
| K0E8E9 | Acyltransferase | | 13 | 0,67 | 0,038 |
| K0E1X6 | Uncharacterized protein FTS_0197 | | 8 | 0,56 | 0,015 |
|  |  | |  |  |  |
