# Supplementary figures and images for: The Multiple Localized Glyceraldehyde-3-Phosphate Dehydrogenase Contributes to the Attenuation of the Francisella tularensis dsbA Deletion Mutant
Source: Front Cell Infect Microbiol. 2017 Dec 11;7:503. doi: 10.3389/fcimb.2017.00503 (PMC5732180; doi:10.3389/fcimb.2017.00503)

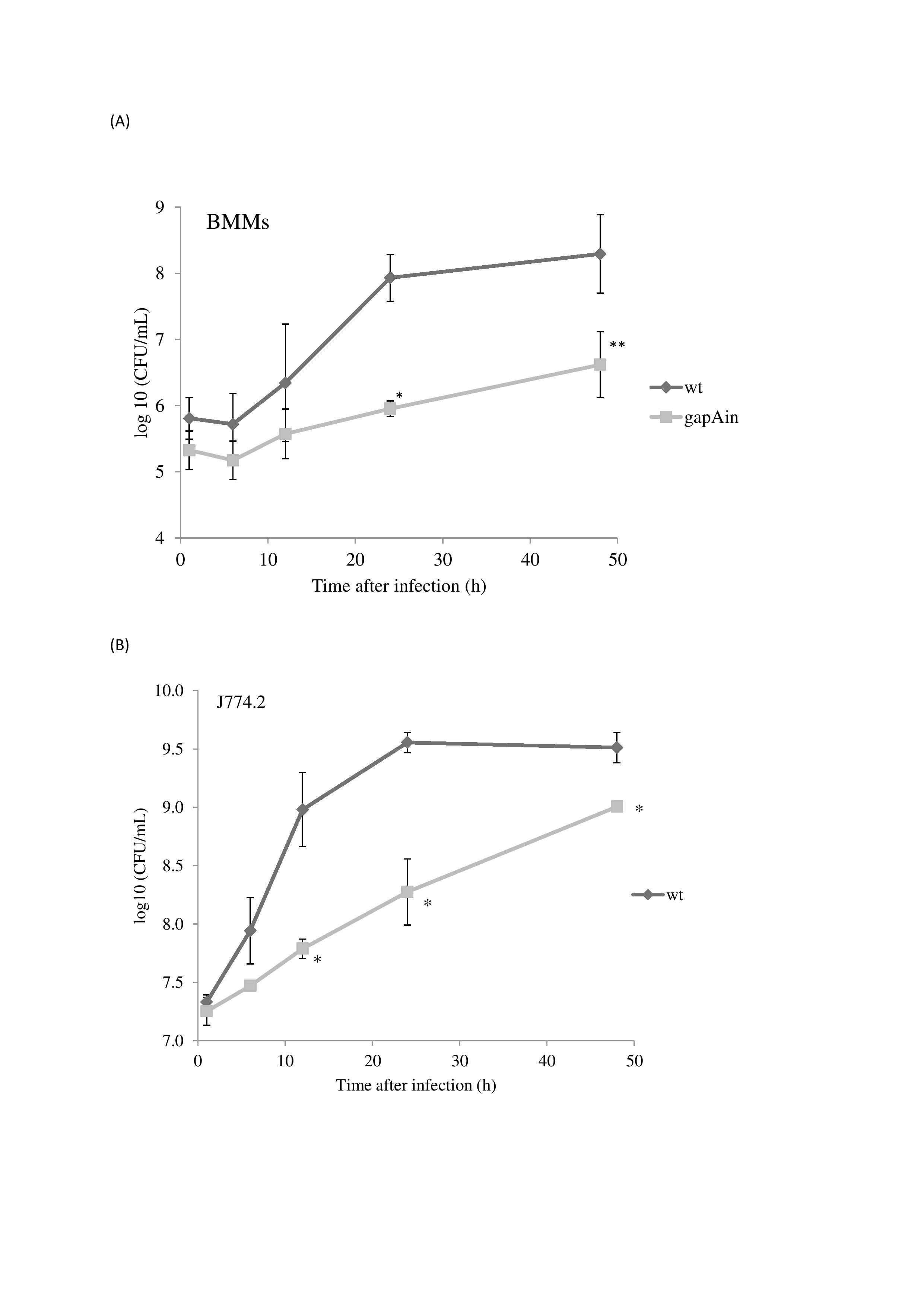

Supplement: Supplementary file 3 [file Image1.JPEG]

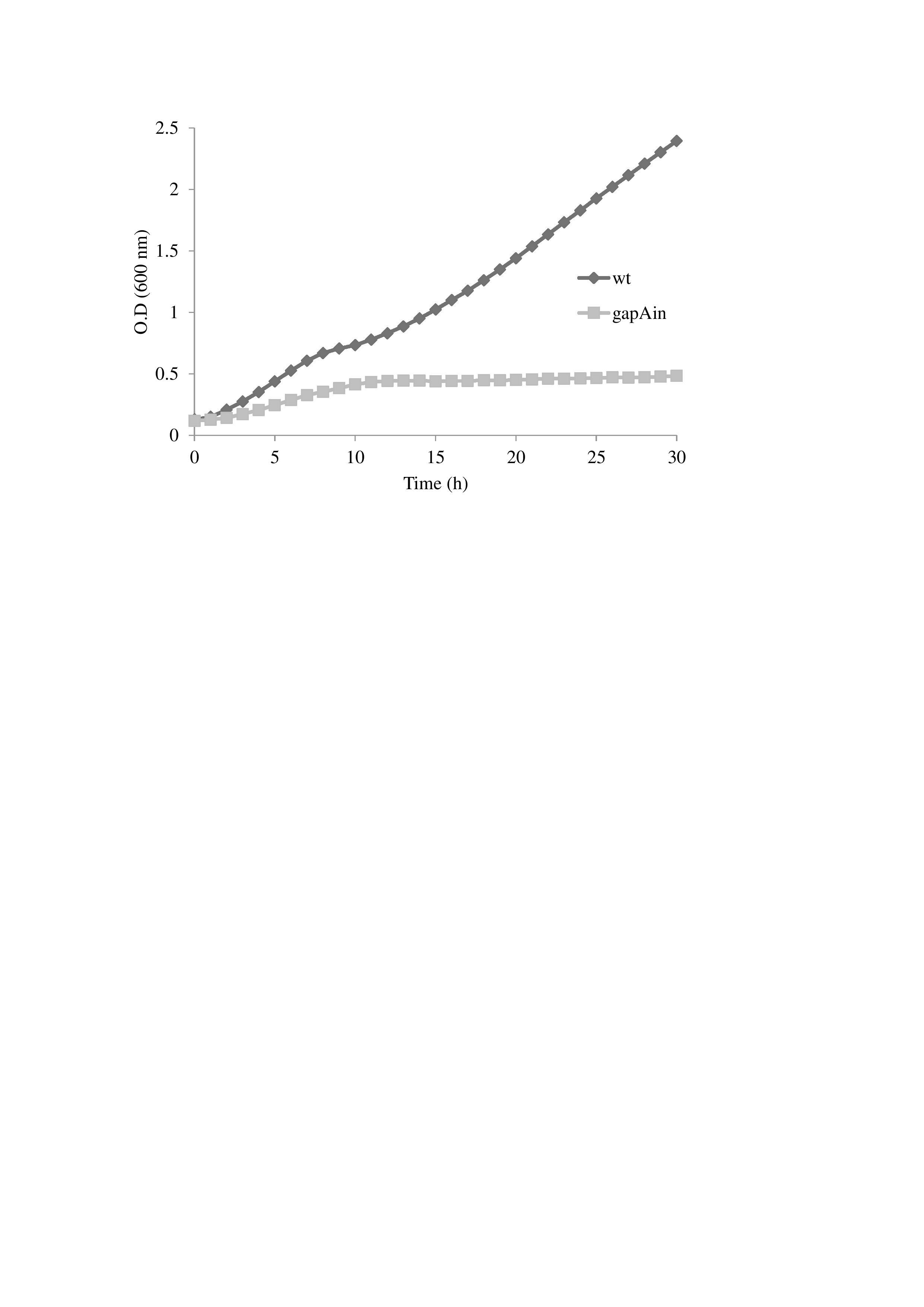

Supplement: Supplementary file 4 [file Image2.JPEG]

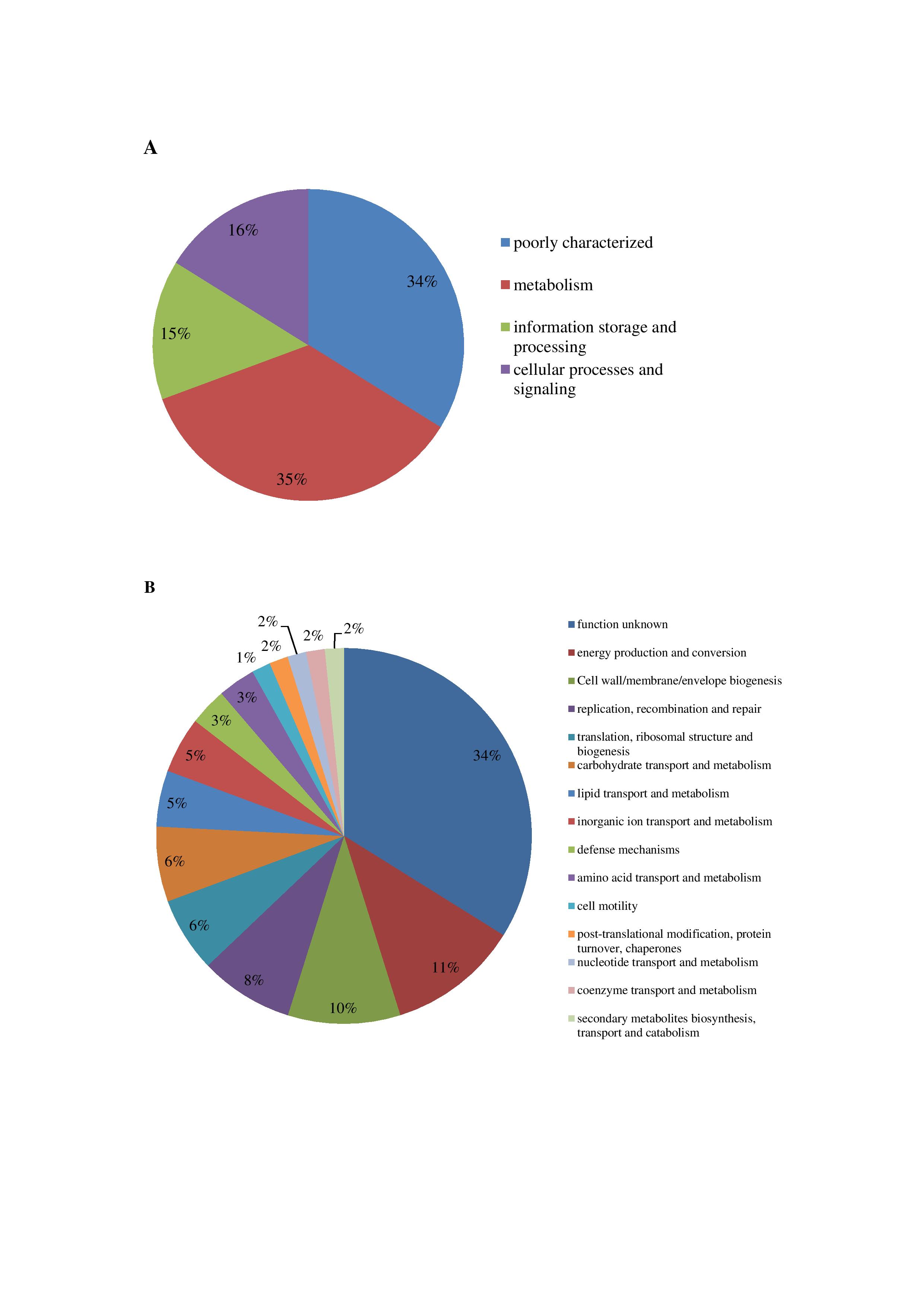

Supplement: Supplementary file 5 [file Image3.JPEG]
