# Supplementary material for: The Multiple Localized Glyceraldehyde-3-Phosphate Dehydrogenase Contributes to the Attenuation of the Francisella tularensis dsbA Deletion Mutant
Source: Front Cell Infect Microbiol. 2017 Dec 11;7:503. doi: 10.3389/fcimb.2017.00503 (PMC5732180; doi:10.3389/fcimb.2017.00503)
Supplement: Supplementary file 6 [file DataSheet1.DOCX]

**Supplementary Material:**

**Construction of TargeTron insertional mutant strains and their application in mouse infection studies**

**TargeTron insertional mutagenesis:** The TargeTron gene knockout system was utilized as described previously (Rodriguez et al., 2009). Targeted sites for insertion and retargeting PCR primers (Supplementary Table 1) were generated using the TargeTron gene knockout system (Sigma-Aldrich, St. Louis, MO, USA). The resulting PCR product was digested (HindIII-BsrgI) and cloned into the *Francisella* targeting vector pKEK1140 (generously provided by Karl Klose, University of Texas at San Antonio, San Antonio, TX, USA). The constructs were introduced into the FSC200 strain by electroporation. Presence of the TargeTron insertion was verified using an intro-specific EBS universal primer combined with gene-specific primers that amplified across the insertion site. Positive clones were incubated in Chamberlain’s medium at 37°C overnight, streaked on McLeod agar, then incubated at 37°C to remove the TargeTron temperature-sensitive plasmid.

Sixteen protein were selected from the SILAC quantitative analysis of fraction enriched in membrane proteins isolated from the strains *F. tularensis* subsp. *holarctica* FSC200 and *F. tularensis* subsp. *holarctica* ΔFTS_1067/FSC200 (ΔdsbA) (Supplementary Table 1) for the construction of relevant TargeTron insertional mutant strains. With the exception of one gene encoding the acyltransferase FTS_0079, we succeeded in creating in total 15 insertion mutants that were tested in the mouse model of infection for potential signs of attenuation.

**Animal studies with TargeTron insertional mutant strains:**

For survival studies, groups of five female BALB/c mice 6–8 weeks old were infected with the TargeTron mutant strains via a subcutaneous route (s.c.) in doses of 3 × 10^2^, 3 × 10^5^, and 3 × 10^7^ CFU/mouse. Control groups of mice were inoculated with sterile saline only. Mice were observed daily for signs of illness or death following infection through a total of 21 days. In case of survival, the mice were challenged at day 21 with 10^2^ CFU/mouse of *F. tularensis* FSC200 s.c. The mice were monitored for survival for the next 21 days. After three weeks, the surviving mice were challenged by s.c. route with the wild-type FSC200 strain in doses of 10^2^ CFU/mouse. The mice were monitored during the next three weeks for disease symptoms or death.

Mice infected with wild-type strain FSC200 and 14 of the prepared insertion mutant strains succumbed to disease 5 days after infection with all the indicated doses. In contrast, all mice immunized with the mutant strain disrupted in the gene encoding glyceraldehyde-3-phosphate dehydrogenase (FTS_1117, *gapA*in) survived all the doses, including even the challenge of 3 × 10^2^ bacteria with the virulent wild-type strain.

**Macrophage proliferation assay** **with gapAin strain**

To generate bone marrow macrophages (BMMs), bone marrow cells were collected from dissected femurs of female BALB/c mice 6–10 weeks old and differentiated into macrophages in Dulbecco’s Modified Eagle Medium (DMEM, Invitrogen) supplemented with 10% fetal bovine serum and 20% L929-conditioned medium for 6–7 days (Celli, 2008). The differentiated BMMs were seeded at a concentration of 5 × 10^5^ cells/well in 24-well plates and infected the next day with *F. tularensis* strains at MOI 50:1 (bacteria/cell). To synchronize the infection, the infected cells were centrifuged at 400 × g for 5 min and incubated at 37°C for 30 min. The extracellular bacteria were then removed by gentamicin treatment (5 µg/mL) for 30 min. For the proliferation assay, the infected BMMs were lysed at selected time points with 0.1% sodium deoxycholate. To determine the number of intracellular bacteria, the lysates were serially diluted and plated on McLeod agar.

The murine monocyte-macrophage cell line J774.2 (ECACC reference no. 85011428) was cultured in DMEM supplemented with 10% fetal bovine serum (Invitrogen). For the cell infection experiments, J774.2 cells were infected with the *F. tularensis* strains at MOI of 500:1 and processed in the same way as described for BMM infection.

The ability of the mutant *gapA*in to replicate within BMMs and J774.2 cells was significantly less than that of the wild-type FSC200 strain (Supplementary Figure 1A and 1B). However, the mutant strain revealed a significant growth defect also in Chamberlain’s chemically defined medium (Supplementary Figure 2). The diminished replication can thus be the consequence of an inherent growth defect.

**SUPPLEMENTARY FIGURE 1 |** Viable counts of intracellular bacteria in BMMs (A) and J774.1 cells (B) infected with *F. tularensis* strains FSC200 and *gapA*in mutant strain at 0, 12, 24, and 48 h after infection. Data are means ± SD of triplicate samples, and the results shown are representatives of three independent experiments. Asterisks indicate statistically significant differences; *, *P* < 0.05; **, *P* < 0.01 (comparing *gapA*in with the wild-type FSC200 strain).

**SUPPLEMENTARY FIGURE 2 |** Growth curves for *F. tularensis* strains FSC200 (wt and *gapA*in) in Chamberlain’s medium incubated at 37°C. Bacterial growth was determined by measuring the OD_600 nm_ in pentaplicate every 10 min for 30 h.

**SUPPLEMENTARY FIGURE 3** **|** Functional annotations of proteins differentially expressed in *dsbA* mutant compared to the wild-type FSC200 strain detected by SILAC quantitative shotgun according to the Clusters of Orthologous Groups of proteins (COG) database (https://www.ncbi.nlm.nih.gov/COG/): (A) COG categories, (B) COG subcategories.
