# Supplementary material for: The Multiple Localized Glyceraldehyde-3-Phosphate Dehydrogenase Contributes to the Attenuation of the Francisella tularensis dsbA Deletion Mutant
Source: Front Cell Infect Microbiol. 2017 Dec 11;7:503. doi: 10.3389/fcimb.2017.00503 (PMC5732180; doi:10.3389/fcimb.2017.00503)
Supplement: Supplementary file 7 [file DataSheet2.docx]

**Captions:**

**SUPPLEMENTARY FIGURE 1: Growth of *gapA*in mutant in the J774.1 and BMM cells.**

**SUPPLEMENTARY FIGURE 2: Growth of *gapA*in mutant in Chamberlain’s medium**.

**SUPPLEMENTARY FIGURE 3: Functional annotations of proteins differentially expressed in *dsbA* mutant.**

**Legend**

**SUPPLEMENTARY FIGURE 1 |** Viable counts of intracellular bacteria in BMMs (A) and J774.1 cells (B) infected with *F. tularensis* strains FSC200 and *gapA*in mutant strain at 0, 12, 24, and 48 h after infection. Data are means ± SD of triplicate samples, and the results shown are representatives of three independent experiments. Asterisks indicate statistically significant differences; *, *P* < 0.05; **, *P* < 0.01 (comparing *gapA*in with the wild-type FSC200 strain).

**SUPPLEMENTARY FIGURE 2 |** Growth curves for *F. tularensis* strains FSC200 (wt and *gapA*in) in Chamberlain’s medium incubated at 37°C. Bacterial growth was determined by measuring the OD_600 nm_ in pentaplicate every 10 min for 30 h.

**SUPPLEMENTARY FIGURE 3** **|** Functional annotations of proteins differentially expressed in *dsbA* mutant compared to the wild-type FSC200 strain detected by SILAC quantitative shotgun according to the Clusters of Orthologous Groups of proteins (COG) database (https://www.ncbi.nlm.nih.gov/COG/): (A) COG categories, (B) COG subcategories.
